# Caffeoyl-*β*-d-glucopyranoside and 1,3-dihydroxy-2-tetracosanoylamino-4-(E)-nonadecene isolated from *Ranunculus muricatus* exhibit antioxidant activity

**DOI:** 10.1038/s41598-019-52166-w

**Published:** 2019-10-30

**Authors:** Farooq Azam, Bashir Ahmad Chaudhry, Hira Ijaz, Muhammad Imran Qadir

**Affiliations:** 1 Department of Pharmacy, University of Agriculture, Faisalabad, Pakistan; 2 Faculty of Pharmacy, Bahauddin Zakariya University, Multan, Pakistan; 3 Institute of Molecular Biology and Biotechnology, Bahauddin Zakariya University, Multan, Pakistan

**Keywords:** Screening, Pharmacology

## Abstract

This study evaluates the antioxidant activity of *Ranunculus muricatus* and isolation and structure elucidation of the active constituents. The aerial parts of the plants were shade dried at room temperature and powdered and extracted with methanol. The free radical scavenging activity was evaluated by 1,1-diphenyl-2-picryl-hydrazyl (DPPH) assay. The percentage scavenging activity was determined based on the percentage of DPPH radical scavenged. Column chromatography was used in order to isolate the active compounds. Spectral techniques UV, IR, ^1^H NMR, ^13^CNMR and HREI-MS were used for the structure elucidation of the isolated compounds. Two isolated compounds, A (caffeoyl-β-d-glucopyranoside) and B (1,3-dihydroxy-2-tetracosanoylamino-4-(E)-nonadecene), exibited a significant antioxidant activity as showed by DPPH radical scavenging method. Percentage inhibition for compound A (at 0.5 mM) was 82.67 ± 0.19 with IC_50_ of 93.25 ± 0.12 (μM), and for compound B (at 0.5 mM) was 69.23 ± 0.19 with IC_50_ of 183.34 ± 0.13 (μM). Quercetin was used as standard control. It was conclued from the present study that caffeoyl-β-d-glucopyranoside and 1,3-dihydroxy-2-tetracosanoylamino-4-(E)-nonadecene isolated from methanol extract of aerial parts of *Ranunculus muricatus* posses antioxidant activity.

## Introduction

Buttercup (*Ranunculus muricatus* L., Family Ranunculaceae) had been used for heart diseases and cancer in folker medicine. Inspection of plant derived drugs included in western pharmacopoeias shows their importance with different pharmacological activity; e.g. quinine from *Cinchona*, reserpine from *Rauwolfia serpentina*, vinblastine and vincristine from *Catharanthus roseus*, taxol from *Taxus brevifolia* and semisynthetic products such as steroidal hormones depends on plant sources for starting material^1^. The Genus *Ranunculus* consists of 600 species. *Ranunculus muricatus*, a species of buttercup known by the common name Spinyfruit buttercup, is distributed in Atlantic and Southern Europe, West and South West Asia, Crimea, Caucasus, Southern Siberia, India and Pakistan^2^.

Tricin 7-O-*β*-d-glucopyranoside, anemonin, *β*-sitosterol, isoscopoletin and protocatechuyl aldehyde were isolated from *Ranunculus muricatus* and have shown antibacterial activity against *Staphylococcus aureus, Micrococcus luteus* and *Escherichia coli, Pseudomonas aeruginosa, Enterobacter cloacae* and *Klebsiella pneumonia*. In particular, tricin 7-O-*β*-d-glucopyranoside showed a potent activity against *Staphylococcus aureus* with MIC value of 0.156 mg/ml^3^.

Search for therapeutically useful herbal antioxidants is a hot subject in the field of medical biology. Recently, many antioxidants have been established; nalewkas, cold brew coffee, *Solanum xanthocarpum*, *Zaleya pentandra*, and *Erythrina superosa* are some of them^4–8^. Caffeoyl derivatives are present in various plants and have been proved to be antioxidants^9–11^.

Objective of the present study was the antioxidant activity evaluation of aerial parts of *Ranunculus muricatus* methanolic extract.

## Results

### Isolation of active constituents

**Figure 1:**
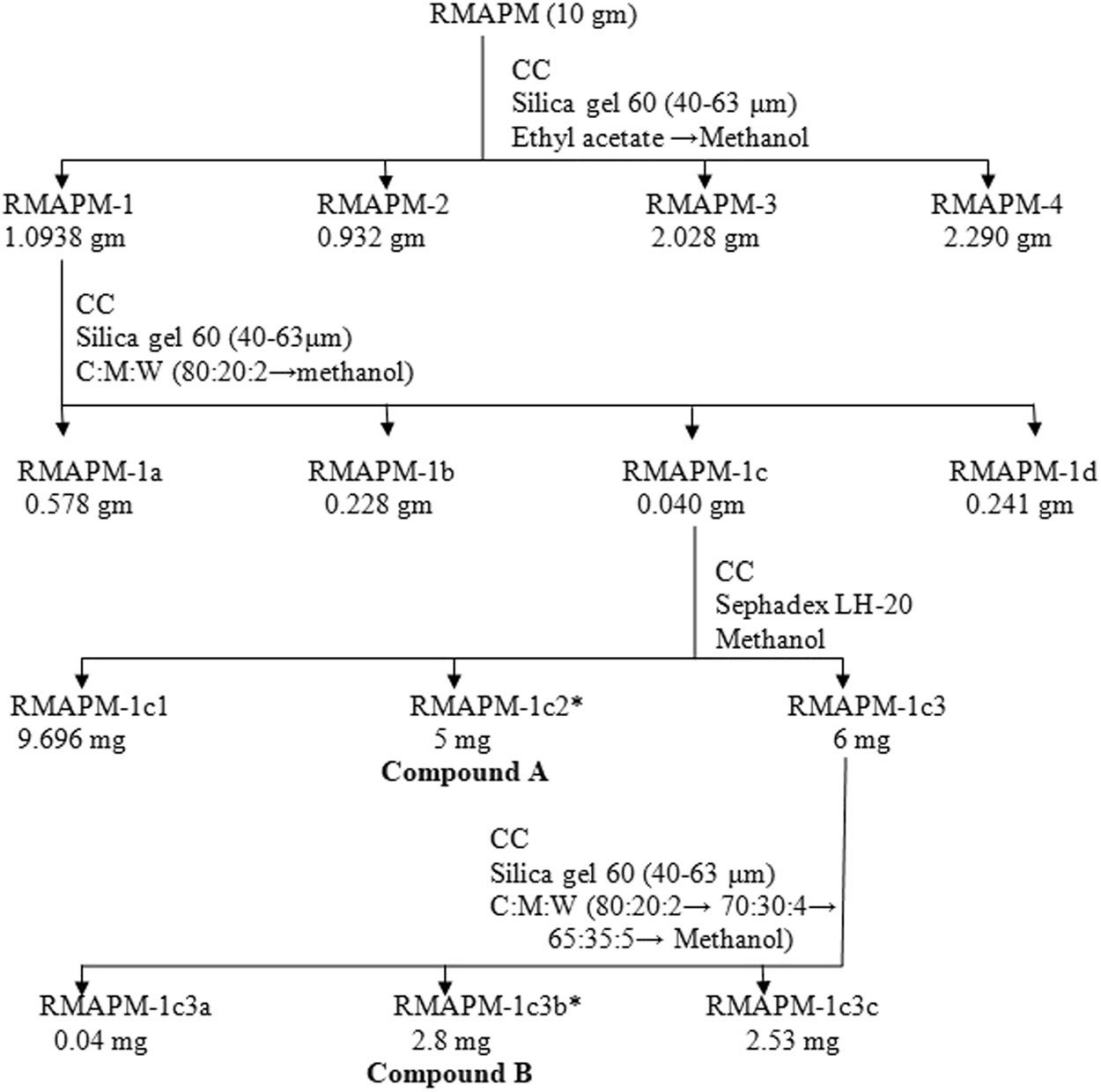
Isolation of compounds A and B from methanol extract of aerial parts of *Ranunculus muricatus*. *The two isolated compounds.

Methanolic extract (10 g) of aerial parts of *Ranunculus muricatus* (RMAPM) was fractionated on column by using silica gel 60 (40–63 μm) as stationary phase, ethyl acetate and methanol was used as mobile phase. Four fractions were obtained (RMAPM_1-4_). The fraction RMAPM-1(1.093 g) was further separated by column chromatography on silica gel 60 (40–63 μm) and chloroform: methanol: water (80:20:2) as mobile phase. Four fractions were obtained (RMAPM-1a to RMAPM-1d). The fractions RMAPM-1c (40 mg) was further fractionated by gel chromatography using Sephadex LH-20 as stationary phase and methanol as mobile phase and four fractions were obtained. The fraction RMAPM-1c2 was found to be pure compound **A** (5 mg). The fraction RMAPM-1c3 (6 mg) was purified by column chromatography on silica gel using stepwise elution to afford compound **B** (RMAPM-1c3b) 2.8 mg. The isolation scheme of compounds **A** and **B** is given in Fig. 1.

### Structural elucidation of isolated compounds

#### Compound A (Caffeoyl-*β*-d-glucopyranoside)

**Figure 2:**
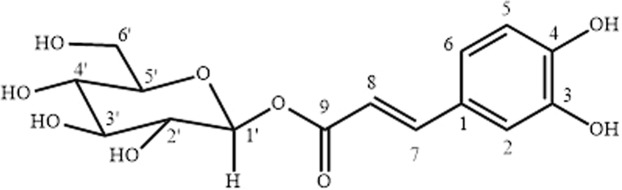
Compound A (Caffeoyl-*β*-d-glucopyranoside).

Chemical structure of Compound A elucidated from the data is given in Fig. 2.

**Physical Data:**

White crystalline solid

**Yield**: 5 mg

**M.P**.: 326 °C

**UV***λ*_max:_ 206 (3.1) nm

**[α]**_**D**_^**25**^: +45(*c* = 0.022, MeOH)

**IR:** (KBr) *ν*_max_ cm^−1^:3423, 1663 and 1600–1400

^**1**^**H-NMR:** (500 MHz)

Proton appeared at *δ* 7.05 (1H, d, *J* = 2.0 Hz, H-2), *δ* 6.96 (1H, dd, *J* = 8.0, 2.0 Hz, H-6), *δ* 6.77 (1H, d, *J* = 8.0 Hz, H-5), *δ* 7.64 (1H,d, *J* = 16 Hz), *δ* 6.29 (1H,d, *J* = 16 Hz), *δ* 5.56 (1H, d, *J* = 8.0 Hz, H-1′), *δ* 3.35–3.46 (1H, m, H-2′), *δ* 3.35–3.46 (1H, m, H-3′), *δ* 3.35–3.46 (1H, m, H-4′), *δ* 3.35–3.46 (1H, m, H-5′), *δ* 3.84(1H, dd, *J* = 12.5, 1.5 Hz, H-6a′) (1H, dd, *J* = 12.0, 5.0 Hz, H-6b′).

^**13**^**C-NMR:** (150 MHz)

Carbon at *δ* 127.57(C-1), *δ* 114.33 (C-2), *δ* 148.4 (C-3), *δ* 149.94 (C-4), *δ* 115.22 (C-6), *δ* 123.26 (C-7), *δ* 146.87 C-7 and *δ* 116.52 (C-8), *δ* 167.79 (C-9), *δ* 95.75(C-1′), *δ* 78.79 (C-2′), *δ* 77.99 (C-3′), *δ* 74.02 (C-4′), *δ* 71.07(C-5′) and *δ* 62.31 (C-6′).

**EI-MS:**

342 (76), 180 (100), 164 (13), 148 (37), 165 (26), 104 (18) and77 (47) *m/z* with (rel. int.)

**HR-EI-MS:**

342.0689 (calculated for C_15_H_18_O_9_, 342.0695)

#### Compound B (1,3-dihydroxy-2-tetracosanoylamino-4-(E)-nonadecene)

**Figure 3:**
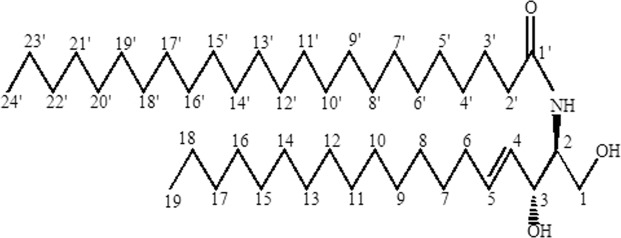
Compound B (1,3-dihydroxy-2-tetracosanoylamino-4-(E)-nonadecene).

Chemical structure of Compound B elucidated from the data is given in Fig. 3.

**Physical Data:**

Colorless amorphous powder

**Yield:** 2.8 mg

**[α]**_**D**_^**25**^**:** −19.1° (*c* 0.021, pyridine)

**IR**; 3300 (O-H), 1660 (C=C), 1642 (C=O) cm^−1^ by KBr methods

**EI-MS:** 284 (31), 269 (16), 225 (21), 195 (12), 169 (10) *m/z* with (rel. int.)

**HR-EI-MS:** 663.6512 (calculated for C_43_H_85_NO_3_, 663.6529)

^**1**^**H-NMR:** (500 MHz)

Proton resonated at *δ* 1.28 (brs, singlet), *δ* 4.17 (dd, *J* = 3.1, 11.1 Hz, H-1), *δ* 4.12 (dd, *J* = 3.1, 11.1 Hz, H-1), *δ* 5.25 (H-5) (dt, *J* = 7, 17 Hz), *δ* 4.96 (H-4) (dd, *J* = 7, 17 Hz), *δ* 0.84 (6H, *t*, *J* = 6.8 Hz, CH_3_-24′, 19), *δ* 4.26 (m, H-3), *δ* 3.96 (m, H-2), *δ* 7.51 (1H, *d*, *J* = 8.7 Hz, NH).

^**13**^**C-NMR:** (125 MHz)

Carbon at *δ* 165.2 (C-1′), *δ* 124.4 (C-4) *δ* 123.9 (C-5) *δ* 76.2 (C-3), *δ* 68.3 (C-1), *δ* 48.6 (C-2) *δ* 31.1, all other methylene in the range of *δ* 29.7–31.4, *δ* 20.5 (C-19), *δ* 14.2 (C-24′).

#### *Antioxidant Activity* (DPPH radical scavenging activities of isolated compounds)

*In vitro* free radical scavenging activities of isolated compounds A & B were performed using the standard techniques. The inhibition (%) at 0.5 mM was measured and IC_50_ (μM) values were calculated. The results are shown in Table 1. Data shows that compounds A and B exhibited a significant antioxidant activity as measured by DPPH radical scavenging method with a percent inhibition value of 82.67 ± 0.19 and 69.23 ± 0.19 respectively at 0.5 mM. Quercetin was used as control.

**Table 1:** Results of DPPH radical scavenging activities of compounds A & B.

Compound	Inhibition (%) at 0.5 mM	IC_50_ (μM)
A	82.67 ± 0.19	93.25 ± 0.12
B	69.23 ± 0.19	183.34 ± 0.13
Standard (Quercetin)	93.21 ± 0.97	16.96 ± 0.14

## Discussion

Different active constituents have been isolated from *Ranunculus* species^12–15^. *Ranunculus* species have been evaluated to have antibacterial^16,17^, cytotoxic^18^, antiviral^19^ and antifungal^20^ activity.

The compound A was obtained as white crystalline solid from the methanol soluble fraction, mp 326 °C. It gave violet colour when treated with FeCl_3_ (recognition test for phenol). The absorption bands in the UV spectrum were observed at 335, 273 and 202 nm while in the IR spectrum showed absorption bands at 3423, 1663 and 1600–1400 cm^−1^. The low resolution EI mass spectrum showed the molecular ion peak at *m/z* 342 while the HR-EIMS showed the [M]^+^ peak at *m/z* 342.0689 which deduced the molecular formula C_15_H_18_O_9_. The ^1^H-NMR spectrum showed signal at substituted phenyl ring (ABX system) at *δ* 7.05 (1H, d, *J* = 2.0 Hz, H-2), *δ* 6.96 (1H, dd, *J* = 8.0, 2.0 Hz, H-6) and *δ* 6.77 (1H, d, *J* = 8.0 Hz, H-5). The signals appeared at *δ* 7.64 (1H, d, *J* = 16 Hz) and *δ* 6.29 (1H, d, *J* = 16 Hz) were assigned due to olefinic protons attached to C-7 and C-8 respectively. The methine protons of glucosidal moiety were observed in the range of *δ* 3.35–3.46 while the anomeric proton resonated as doublet at *δ* 5.56 (1H, d, *J* = 8.0 Hz, H-1′). The ^13^C NMR (BB and DEPT) spectra showed the 15 carbon signals consisting of one methylene, ten methine and four quaternary carbon atoms. The ^13^C-NMR spectrum showed characteristic pattern of cinnamoyl moiety while the signals at *δ* 95.75 and *δ* 79.2–62.3 were of glucose moiety. On the basis of spectral data compound A could be identified as caffeoyl- *β*-D-glucopyranoside^21^.

Compound B was obtained as a colorless amorphous solid belongs to ceramide family. Its IR spectrum gives absorptions at 3300 cm^−1^ for a hydroxyl moiety, 1642 cm^−1^ for a carbonyl group. EIMS gives M^+^ ion peak at *m*/*z* 663 (calculated for C_43_H_85_NO_3_, 663.6529) corresponding to the molecular formula C_43_H_85_NO_3._ The large numbers of methylene groups at *δ* 1.28 were observed in ^1^H-NMR spectrum. It further showed nonequivalent methylene proton (H-1) signals at *δ* 4.17 (dd, *J* = 3.1, 11.1 Hz) and *δ* 4.12 (dd, *J* = 3.1, 11.1 Hz), two olefinic proton signals at *δ* 5.25 (H-5) (dt, *J* = 7, 17 Hz) and *δ* 4.96 (H-4) (dd, *J* = 7, 17 Hz) and two methine protons at *δ* 4.26 (m, H-3) and *δ* 3.96 (m, H-2). The large coupling constants (*J* = 17 Hz) of two methine protons at *δ* 5.25 and *δ* 4.96 revealed the (E) configuration of the double bond. The ^13^NMR DEPT spectrum showed five methine carbon groups resonated at *δ* 124.8, 123.1, 76.2, 48.6, 31.1, one oxy methylene carbons appeared at *δ* 68.3 and two methyl carbons appeared at *δ* 20.5 and *δ*14.2. There connectivities were determined by using 2D NMR techniques including HMBC and HMQC. On the basis of reported data compound B could be identified as 1,3-dihydroxy-2-tetracosanoylamino-4-(E)-nonadecene^22^.

Ranunculaceae are generally well known for their antioxidant properties. Many active compounds have been isolated from these species that have been evidenced to posses’ antioxidant activity*. Ranunculus acris, Ranunculus platanifolius, Ranunculus repens*, and *Ranunculus serpens* were characterized by a high percentage of hexadecanoic acid, followed by phytol, octadecadienoic and octadecatrienoic acids, which have antioxidant activities^23,24^. Caffeoyl derivatives have been demonstrated to have antioxidant properties. 3,4-di-*O*-caffeoylquinic acid, methyl 3,4-di-*O*-caffeoyl quinate, 3,5-di-*O*-caffeoylquinic acid, methyl 3,5-di-*O*-caffeoyl quinate, 4,5-di-*O*-caffeoylquinic acid and methyl 4,5-di-*O*-caffeoyl quinate present in *Dipsacus asper* showed antioxidant activity^11^. Caffeoyl malate anhydride and its derivative also showed antioxidant activity^25^. Caffeoylaltraric acid and caffeoylquinic acid present in *Smallanthus sonchifolius* also showed antioxidant property^9^. Therefore, presence of caffeoyl derivative in *Ranunculus muricatus* gave a clue to have antioxidant activity and finaly, the present study verified the anti-oxidant activity of *Ranunculus muricatus* by using DPPH radical scavenging method.

## Conclusion

It was concluded from the present study that caffeoyl-β-d-glucopyranoside and 1,3-dihydroxy-2-tetracosanoylamino-4-(E)-nonadecene isolated from methanol extract of aerial parts of *Ranunculus muricatus* posses antioxidant activity.

## Materials and Methods

### Plant material

The plant material of *Ranunculus muricatus* was collected from the fresh water of the Lower Bari Doab Canal flowing in the area of Khanewal in the month of April 2012. The plant was identified by Prof. Dr. Altaf Ahmad Dasti and Dr. Zafarullah Zafar, Institute of Pure and Applied Biology, Bahauddin Zakariya University, Multan, Pakistan. The voucher specimen has been deposited in herbarium with reference No. R.R. STEWART 271-7.

### Extraction

The plants parts were shade dried at room temperature and powdered and extracted with methanol at room temperature for 24 hours. The extracts were concentrated under vacuum on Rota vapor model No. Buchi-rotavapor R.200.

### Antioxidant activity

The free radical scavenging activity was measured by 1,1-diphenyl-2-picryl-hydrazyl (DPPH) assay^7^. The DPPH stock solution was prepared by dissolving 20 mg DPPH in 100 ml 95% methanol. This stock solution was stored at −20 °C until needed not more than 10 days. DPPH working solution was prepared by diluting the stock of DPPH solution by adding methanol and absorbance was adjusted about 0.980 ± 0.02 at wave length 517 nm using the spectrophotometer. 3 ml aliquot of this working solution mixed with 100 µl of the plant samples at five different varying concentrations (4–322 µg/ml). The solutions in the test tubes shacked well and put in dark for 15 minutes at room temperature. Then again the absorbance was measured at 517 nm. The percentage scavenging activity was determined based on the percentage of DPPH radical scavenged by using the following equation.

$${\rm{Scavenging}}\,{\rm{effect}}( \% )=[{\rm{control}}\,{\rm{absorbance}}-{\rm{sample}}\,\mathrm{absorbance}/\mathrm{control}\,{\rm{absorbance}}]\times 100$$

### Spectral analysis

Column chromatography was used for the active compounds isolation. Ultraviolet (UV) spectras (MeOH, solvents) were recorded on Perkin Elmer Lambda-25 spectrophotometer. Infrared (IR) spectra were measured on Alpha Bruker Infrared instruments. Proton nuclear magnetic resonance (^1^H-NMR) spectra were recorded in CD_3_OD and CDCl_3_ using TMS as internal standard at 300 MHz, 400 MHz, 500 MHz and 600 MHz on Bruker NMR spectrometers. The ^13^C-NMR spectra at (75, 100, 125 and 150 MHz) were also recorded using same instruments in same solvents as in ^1^H-NMR. The 2D NMR (HMBC, HSQC) spectra were recorded in CD_3_OD, and CDCl_3_ at 300 MHz, 400 MHz, 500 MHz and 600 MHz on the same instruments.

Low resolution EIMS spectra were recorded on a Finnigan MAT 311 with MassPec data system. Peak matching, fields desorption (FD) and field ionization (FI) were performed on the Finnigan MAT 312 mass spectrometer. Jeol JMS (HX 110) MS were used for the measurement of HRMS.

### Statistical analysis

The statistics applied was the student *t*-test and *p* < 0.05 was considered as significant.

### Ethical approval

Experimental protocols were approved by the Institutional Ethical Review Committee. Furthermore, all methods were performed in accordance with the relevant guidelines and regulations.

## Supplementary information

Supplementary Dataset 1

## Data Availability

All the data is available in the manuscript.
