# Supplementary material for: Caffeoyl-β-d-glucopyranoside and 1,3-dihydroxy-2-tetracosanoylamino-4-(E)-nonadecene isolated from Ranunculus muricatus exhibit antioxidant activity
Source: Sci Rep. 2019 Oct 30;9:15613. doi: 10.1038/s41598-019-52166-w (PMC6821768; doi:10.1038/s41598-019-52166-w)
Supplement: Supplementary file 1 — Supplementary Dataset 1 [file 41598_2019_52166_MOESM1_ESM.pdf]

Caffeoyl-β-D-glucopyranoside and 1,3-dihydroxy-2-tetracosanoylamino-4-(E)-nonadecene isolated from *Ranunculus muricatus* exhibit antioxidant activity

Farooq Azam, Bashir Ahmad Chaudhry, Hira Ijaz, Muhammad Imran Qadir*

*Correspondence Author: mrimranqadir@hotmail.com

SUPPORTING MATERIAL

Spectral analysis for the isolated compounds: RMH, Code for Compound A that was confirmed as Caffeoyl-β-d-glucopyranoside, and RMI, Code for Compound B that was confirmed as 1,3-dihydroxy-2-tetracosanoylamino-4-(E)-nonadecene.

RMH-BB

DR.SHAKIL/RMH
BB

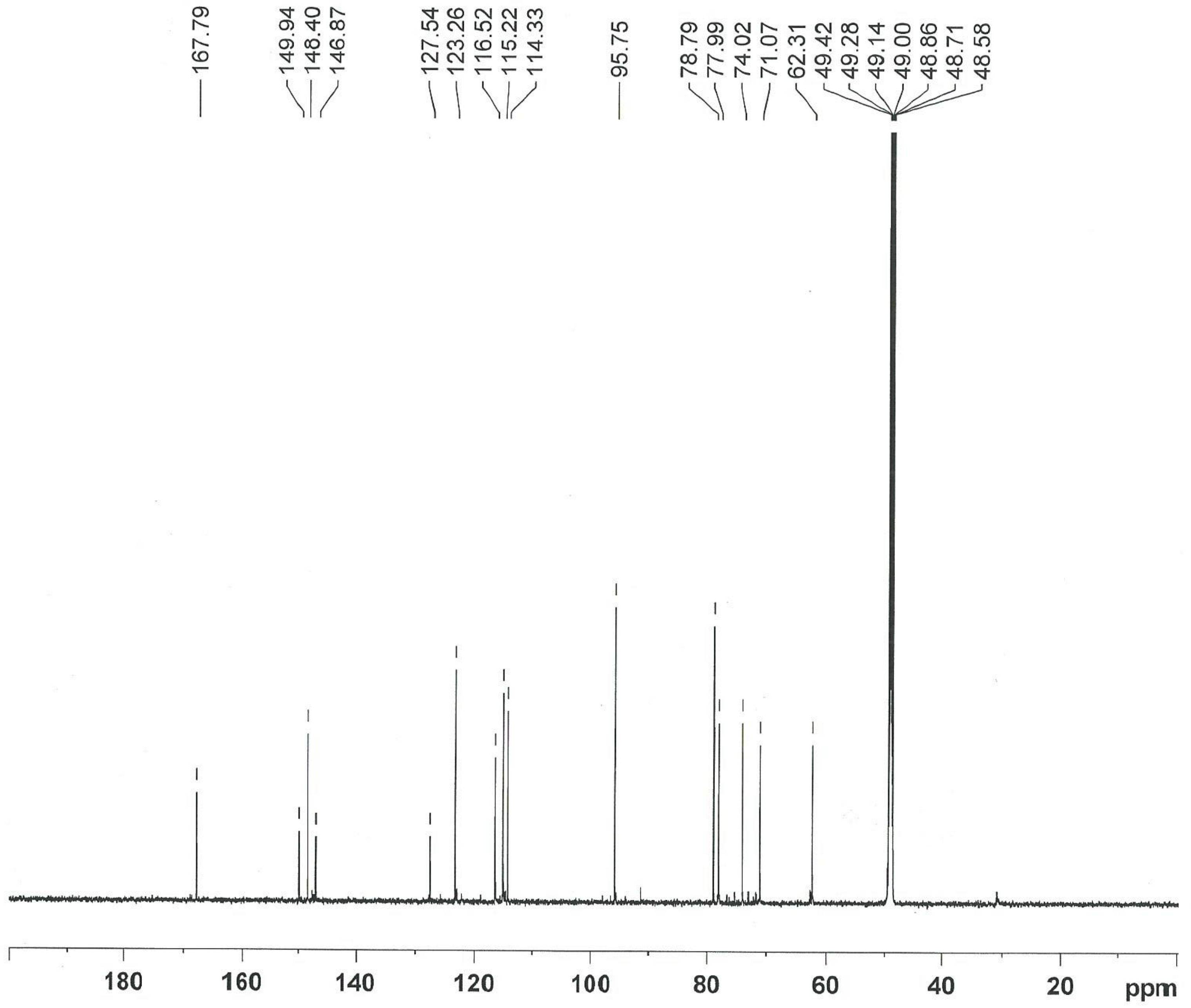

AVANCE AV-600
CRYO PROBE
LAB NO: 108

NAME	dec02-14
EXPNO	4
PROCNO	1
Date_	20141202
Time	12.41
INSTRUM	spect
PROBHD	5 mm CPTCI 1H-
PULPROG	zgpg
TD	32768
SOLVENT	MeOD
NS	4188
DS	2
SWH	35971.223 Hz
FIDRES	1.097755 Hz
AQ	0.4555391 sec
RG	32768
DW	13.900 usec
DE	6.50 usec
TE	298.0 K
D1	1.50000000 sec
D11	0.03000000 sec
TD0	8

======== CHANNEL f1 ========

NUC1	13C
P1	15.40 usec
PL1	1.00 dB
PL1W	83.60149384 W
SFO1	150.9453107 MHz

======== CHANNEL f2 ========

CPDPRG2	waltz16
NUC2	1H
PCPD2	65.00 usec
PL2	3.30 dB
PL12	22.06 dB
PL13	27.00 dB
PL2W	9.16420078 W
PL12W	0.12192553 W
PL13W	0.03909260 W
SFO2	600.2336014 MHz
SI	16384
SF	150.9277423 MHz
WDW	EM
SSB	0
LB	1.00 Hz
GB	0
PC	1.00

DR.SHAKIL/RMH
BB

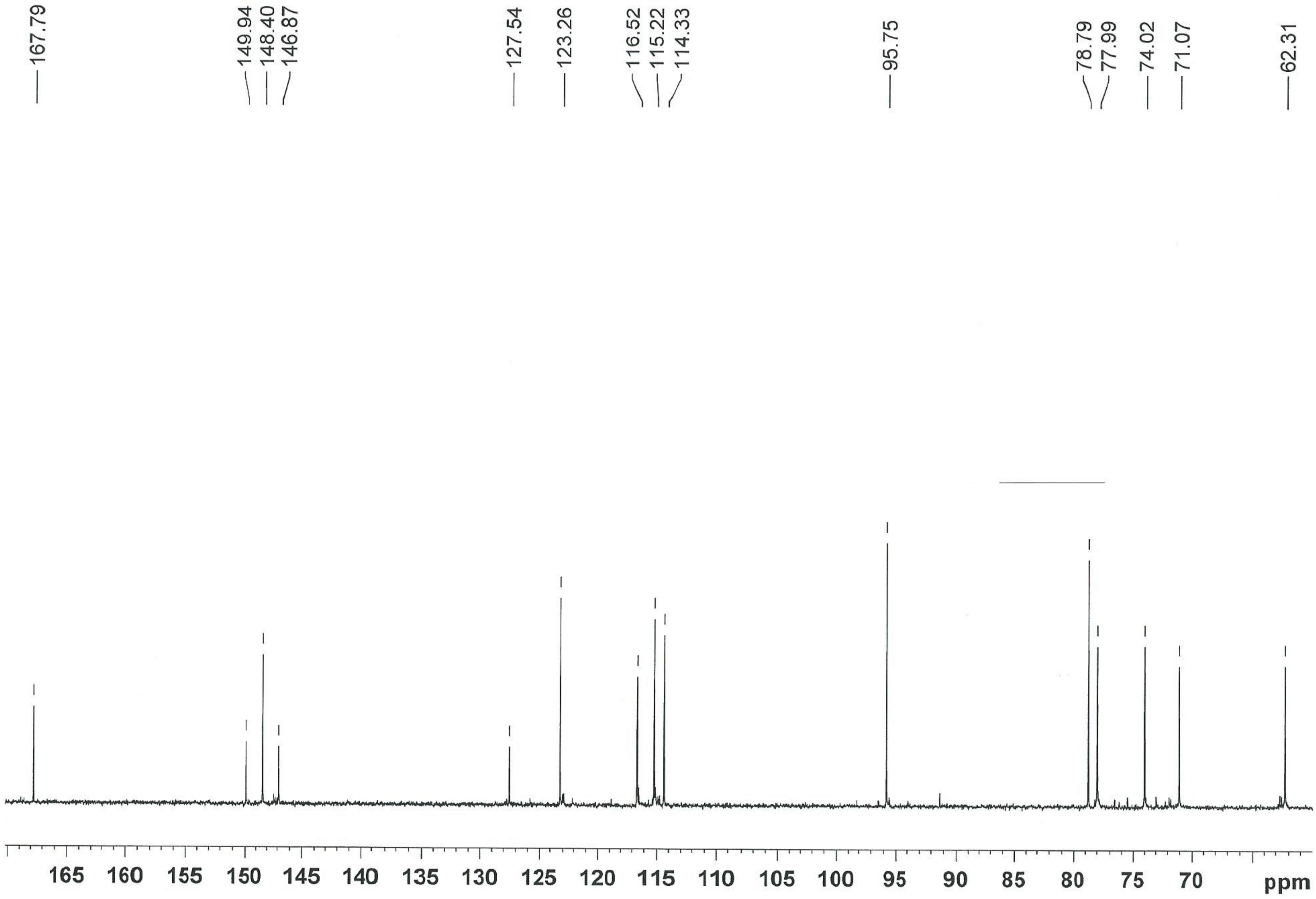

DR.SHAKIL/RMH
DEPT90

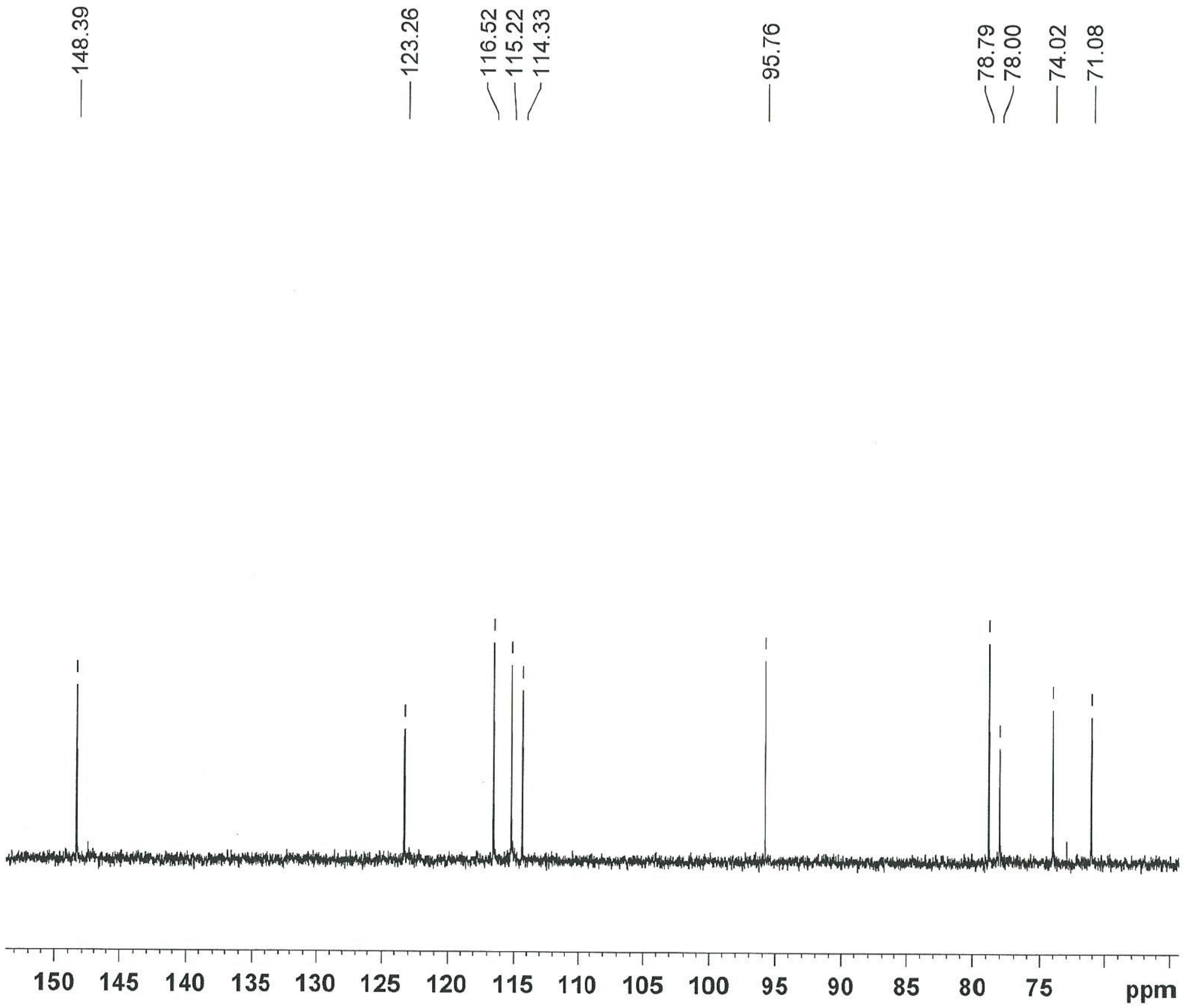

AVANCE AV-600
CRYO PROBE
LAB NO: 108

NAME	dec02-14	
EXPNO	6	
PROCNO	1	
Date_	20141202	
Time	15.03	
INSTRUM	spect	
PROBHD	5 mm CPTCI 1H-	
PULPROG	deptsp90	
TD	32768	
SOLVENT	MeOD	
NS	299	
DS	2	
SWH	30303.031	Hz
FIDRES	0.924775	Hz
AQ	0.5407385	sec
RG	32768	
DW	16.500	usec
DE	6.50	usec
TE	298.0	K
CNST2	145.0000000	
D1	1.50000000	sec
D2	0.00344828	sec
D12	0.00002000	sec
TD0	3	

======== CHANNEL f1 ========

NUC1	13C	
P1	15.40	usec
P12	2000.00	usec
PL0	120.00	dB
PL1	1.00	dB
PL0W	0.00000000	W
PL1W	83.60149384	W
SFO1	150.9430468	MHz
SP2	5.40	dB
SPNAM2	Crp60comp.4	
SPOAL2	0.500	
SPOFFS2	0.00	Hz

======== CHANNEL f2 ========

CPDPRG2	waltz16	
NUC2	1H	
P3	7.50	usec
P4	15.00	usec
PCPD2	65.00	usec
PL2	3.30	dB
PL12	22.06	dB
PL2W	9.16420078	W
PL12W	0.12192553	W
SFO2	600.2324009	MHz
SI	16384	
SF	150.9277423	MHz
WDW	EM	
SSB	0	
LB	1.00	Hz
GB	0	
PC	1.00	

RMH-HMBC

DR.SHAKIL/ RMH
HMBC

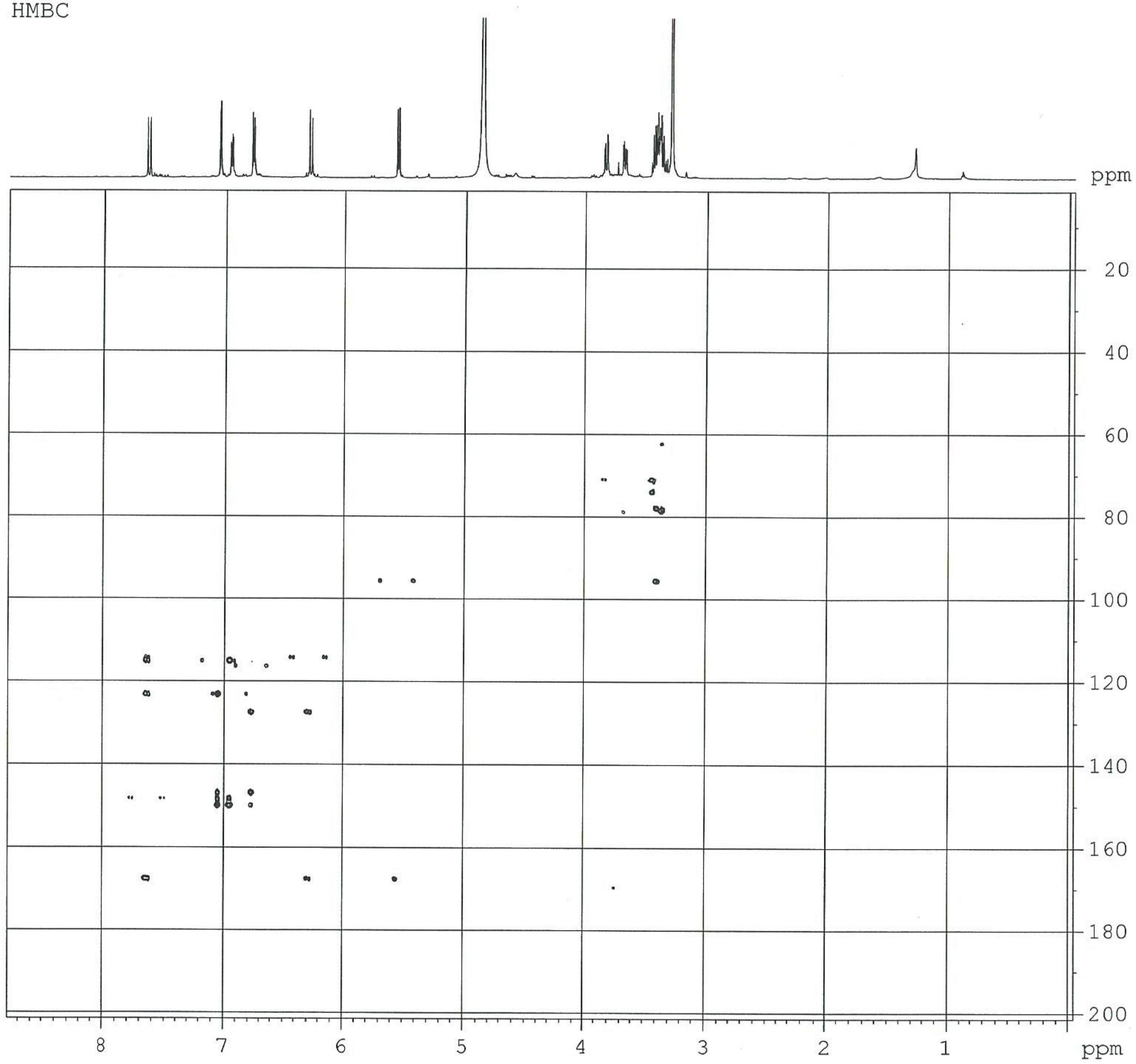

AVANCE AV-600
CRYO PROBE
LAB NO: 108

NAME	dec04-14	
EXPNO	3	
PROCNO	1	
Date_	20141204	
Time	16.24	
INSTRUM	spect	
PROBHD	5 mm CPTCI 1H-	
PULPROG	hmbcgplpndqf	
TD	4096	
SOLVENT	MeOD	
NS	64	
DS	8	
SWH	5296.610	Hz
FIDRES	1.293118	Hz
AQ	0.3868068	sec
RG	41285.1	
DW	94.400	usec
DE	6.50	usec
TE	298.0	K
CNST2	145.0000000	
CNST13	13.0000000	
D0	0.00000300	sec
D1	2.00000000	sec
D2	0.00344828	sec
D6	0.03846154	sec
D16	0.00015000	sec
IN0	0.00001440	sec
======== CHANNEL f1 ========		
NUC1	1H	
P1	7.20	usec
P2	14.40	usec
PL1	3.30	dB
PL1W	9.16420078	W
SFO1	600.2326410	MHz
======== CHANNEL f2 ========		
NUC2	13C	
P3	15.40	usec
PL2	1.00	dB
PL2W	83.60149384	W
SFO2	150.9453107	MHz
====== GRADIENT CHANNEL =====		
GPNAM1	SINE.100	
GPNAM2	SINE.100	
GPNAM3	SINE.100	
GPZ1	50.00	%
GPZ2	30.00	%
GPZ3	40.10	%
P16	2000.00	usec
ND0	2	
TD	256	
SFO1	150.9453	MHz
FIDRES	135.614929	Hz
SW	230.000	ppm
FnMODE	QF	
SI	1024	
SF	600.2300156	MHz
WDW	SINE	
SSB	0	
LB	0.00	Hz
GB	0	
PC	1.40	
SI	1024	
MC2	QF	
SF	150.9277423	MHz
WDW	SINE	
SSB	0	
LB	0.00	Hz
GB	0	

DR.SHAKIL/ RMH
HMBC

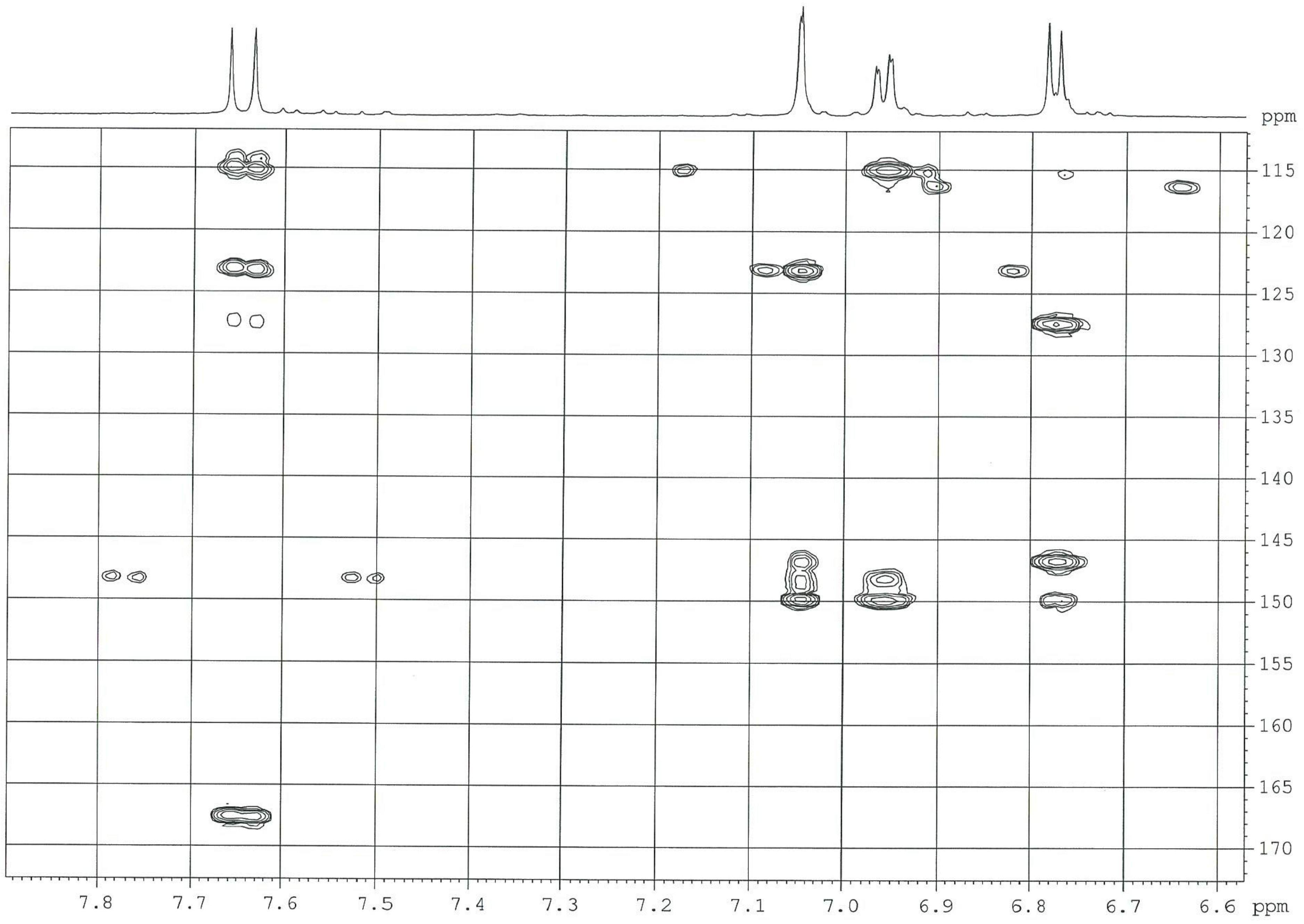

DR.SHAKIL/ RMH
HMBC

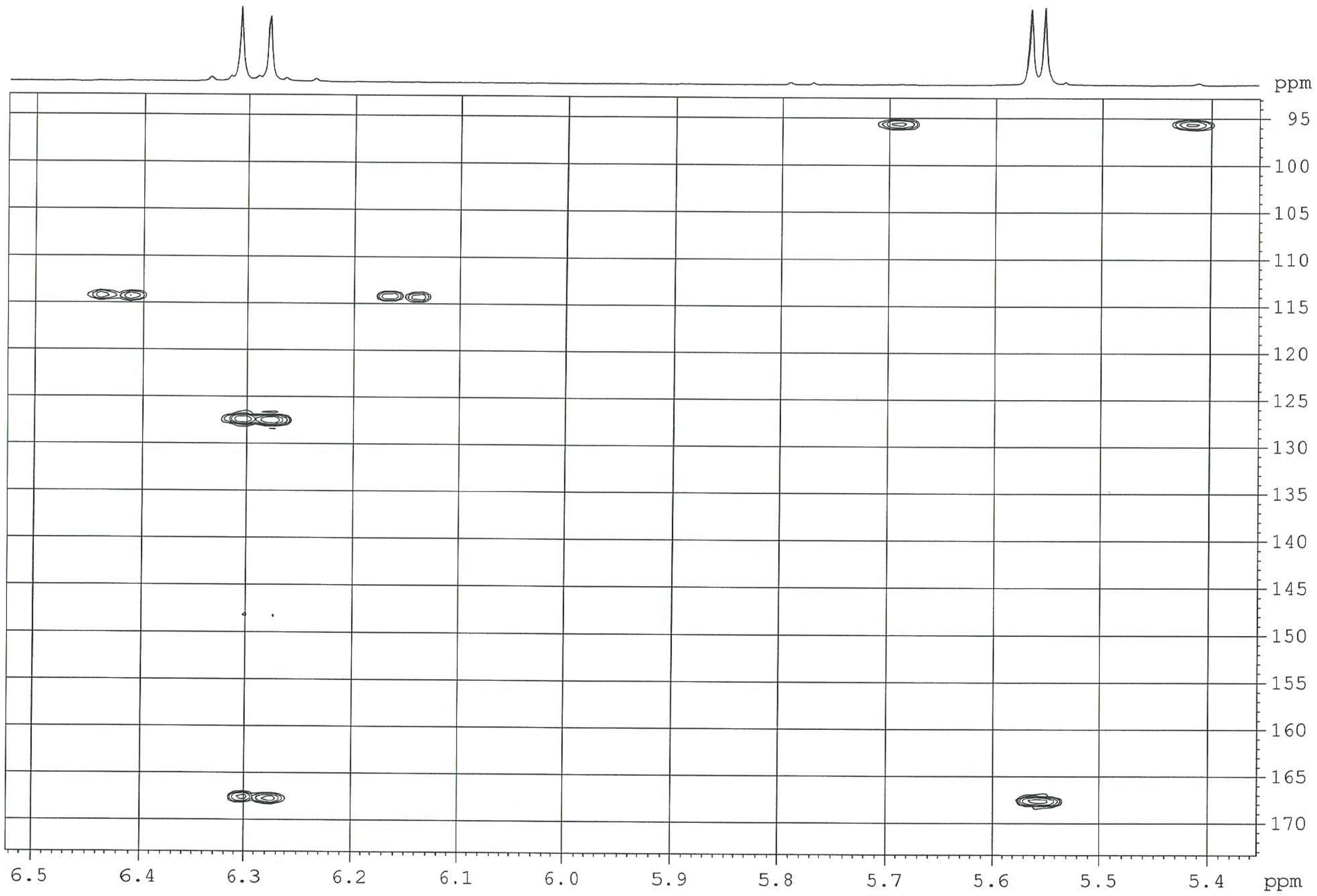

DR.SHAKIL/ RMH
HMBC

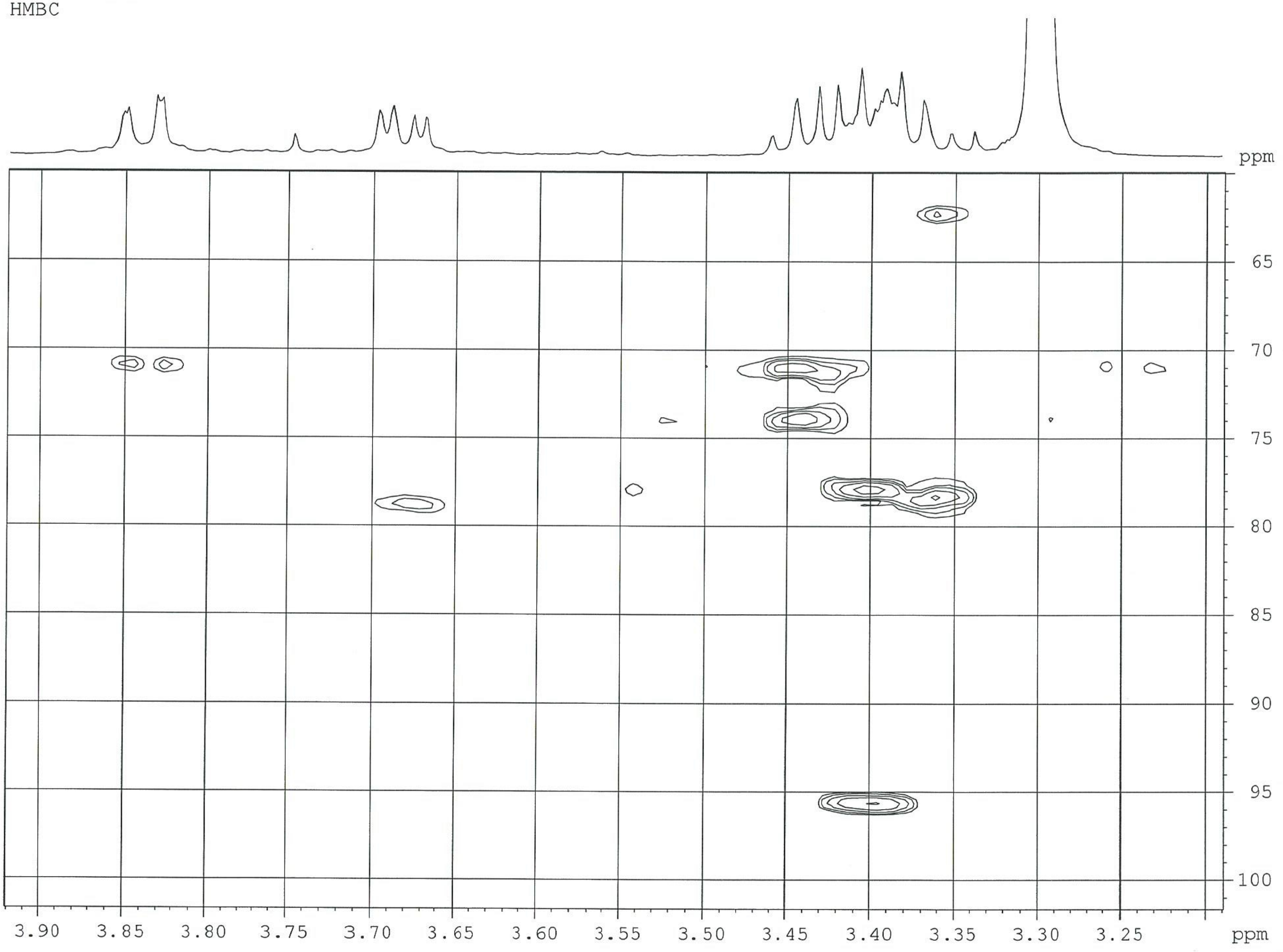

DR.SHAKIL/ RMH
HMBC

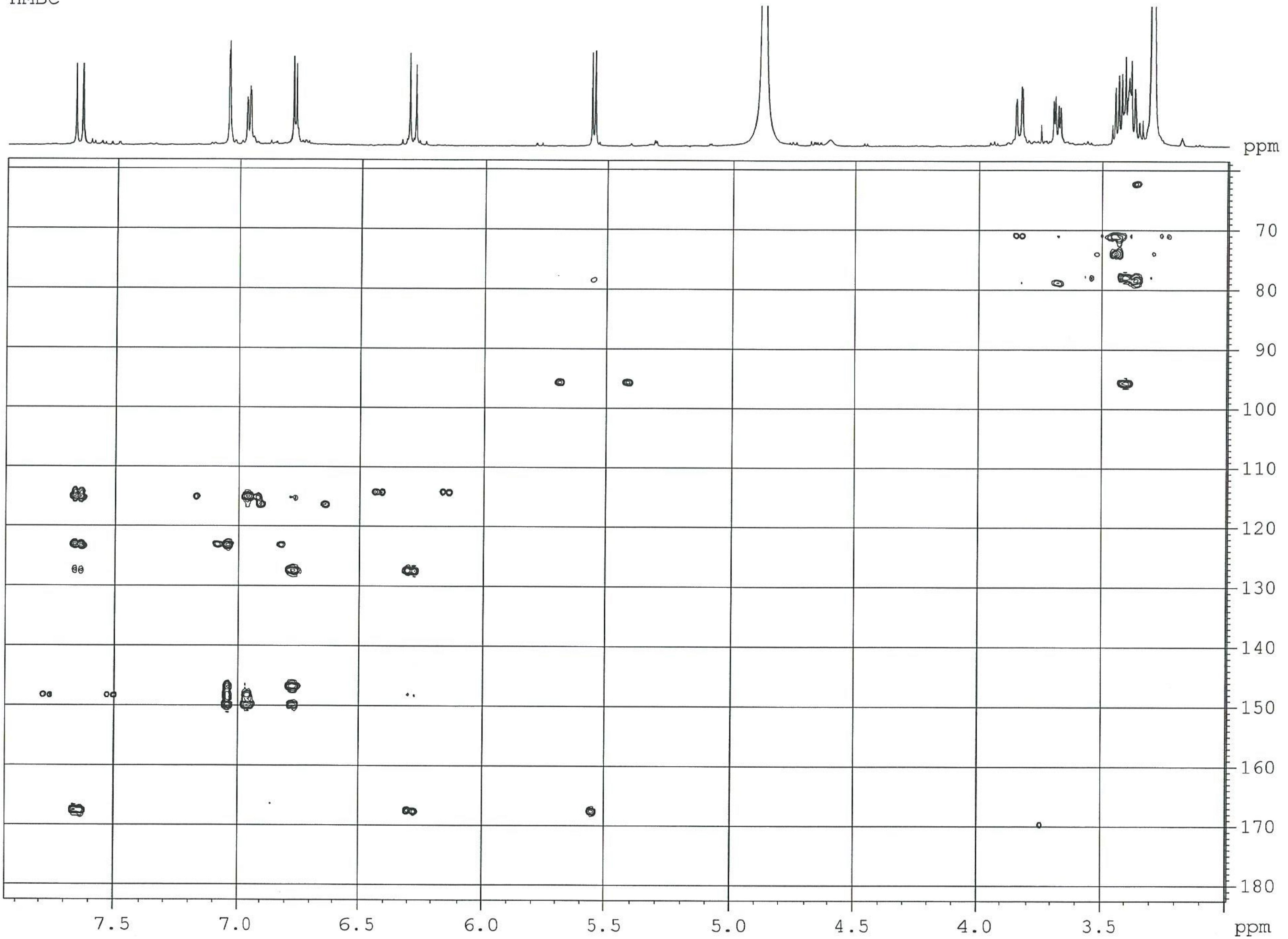

RMH-HSQC

DR.SHAKIL/ RMH
ICCBS/U.O.K
HSQC

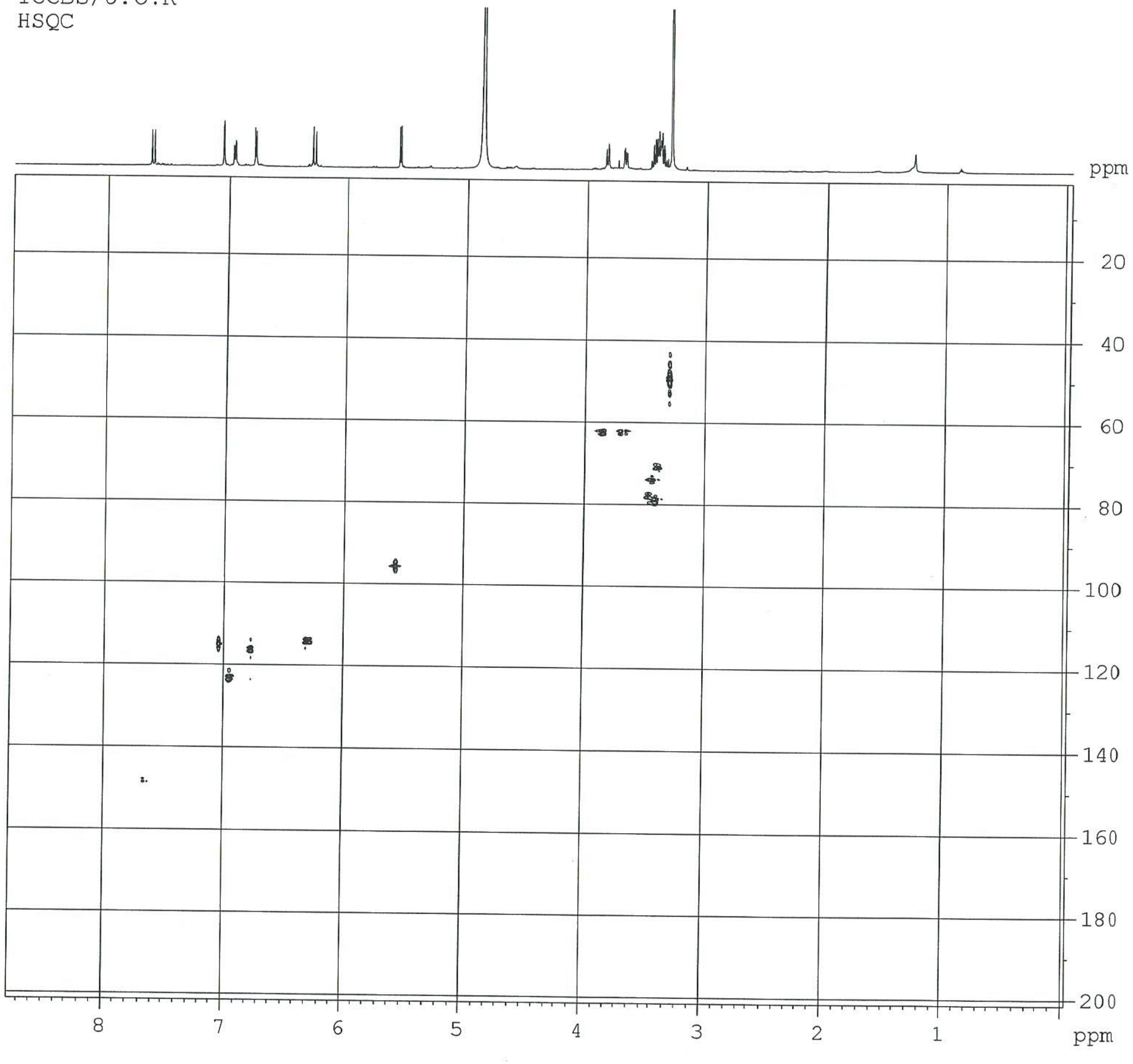

AVANCE AV-600
CRYO PROBE
LAB NO: 108

NAME	dec04-14	
EXPNO	2	
PROCNO	1	
Date_	20141204	
Time	11.32	
INSTRUM	spect	
PROBHD	5 mm CPTCI 1H-	
PULPROG	hsqcetgpsi	
TD	1024	
SOLVENT	MeOD	
NS	32	
DS	8	
SWH	5296.610	Hz
FIDRES	5.172471	Hz
AQ	0.0968100	sec
RG	29193	
DW	94.400	usec
DE	6.50	usec
TE	298.0	K
CNST2	145.0000000	
D0	0.00000300	sec
D1	2.00000000	sec
D4	0.00172414	sec
D11	0.03000000	sec
D13	0.00000400	sec
D16	0.00015000	sec
D24	0.00110000	sec
IN0	0.00001655	sec
ZGOPTNS		
======== CHANNEL f1 ========		
NUC1	1H	
P1	7.20	usec
P2	14.40	usec
P28	0.50	usec
PL1	3.30	dB
PL1W	9.16420078	W
SFO1	600.2326410	MHz
======== CHANNEL f2 ========		
CPDPRG2	garp	
NUC2	13C	
P3	15.40	usec
P4	30.80	usec
PCPD2	61.00	usec
PL2	1.00	dB
PL12	13.00	dB
PL2W	83.60149384	W
PL12W	5.27489758	W
SFO2	150.9430468	MHz
====== GRADIENT CHANNEL =====		
GPNAM1	SINE.100	
GPNAM2	SINE.100	
GPZ1	80.00	%
GPZ2	20.10	%
P16	2000.00	usec
ND0	2	
TD	256	
SFO1	150.943	MHz
FIDRES	117.924255	Hz
SW	200.000	ppm
FnMODE	Echo-Antiecho	
SI	1024	
SF	600.2300156	MHz
WDW	QSINE	
SSB	2	
LB	0.00	Hz
GB	0	
PC	4.00	
SI	1024	
MC2	echo-antiecho	
SF	150.9277423	MHz
WDW	QSINE	
SSB	2	
LB	0.00	Hz
GB	0	

DR.SHAKIL/ RMH
ICCBS/U.O.K
HSQC

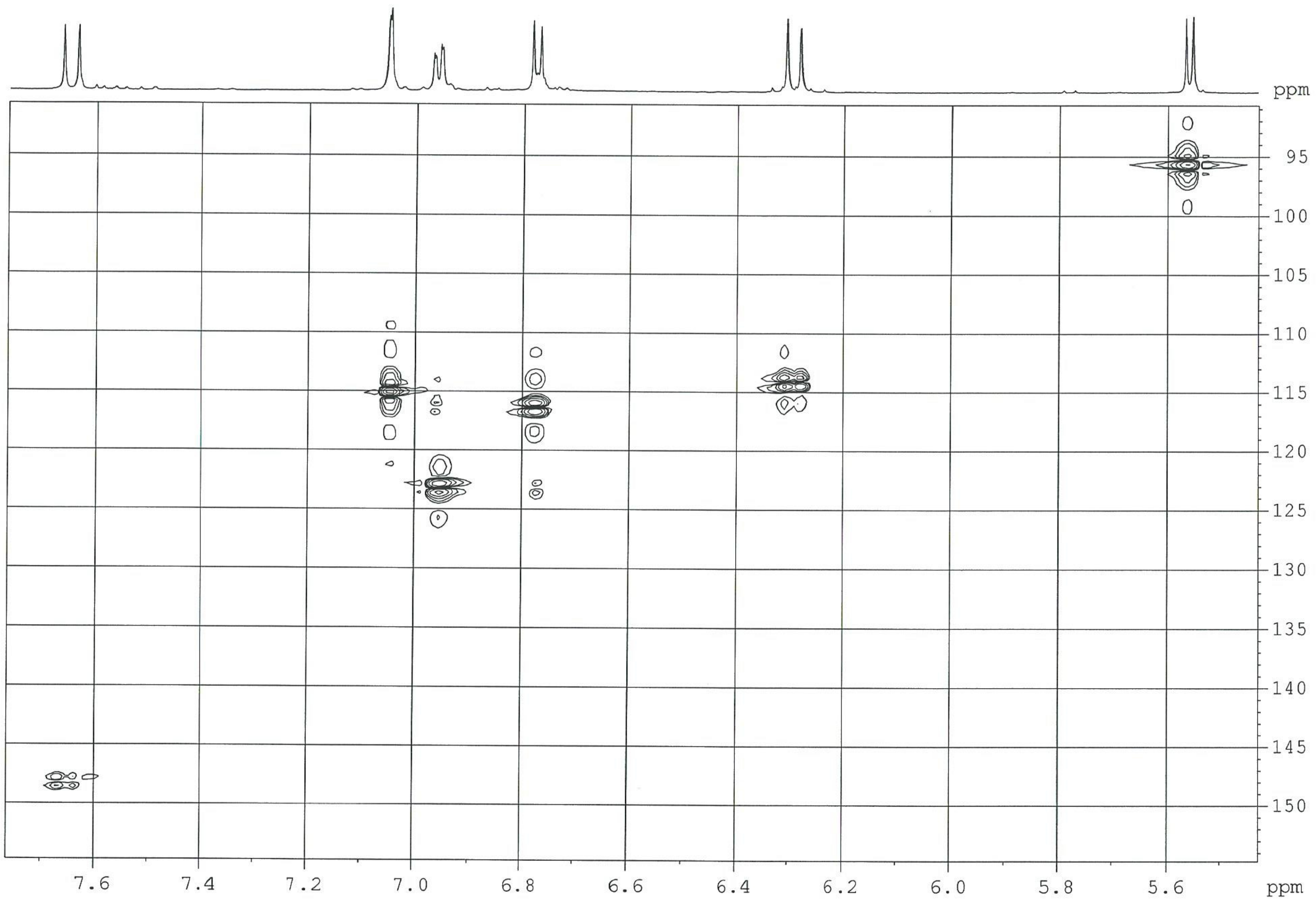

DR.SHAKIL/ RMH
ICCBS/U.O.K
HSQC

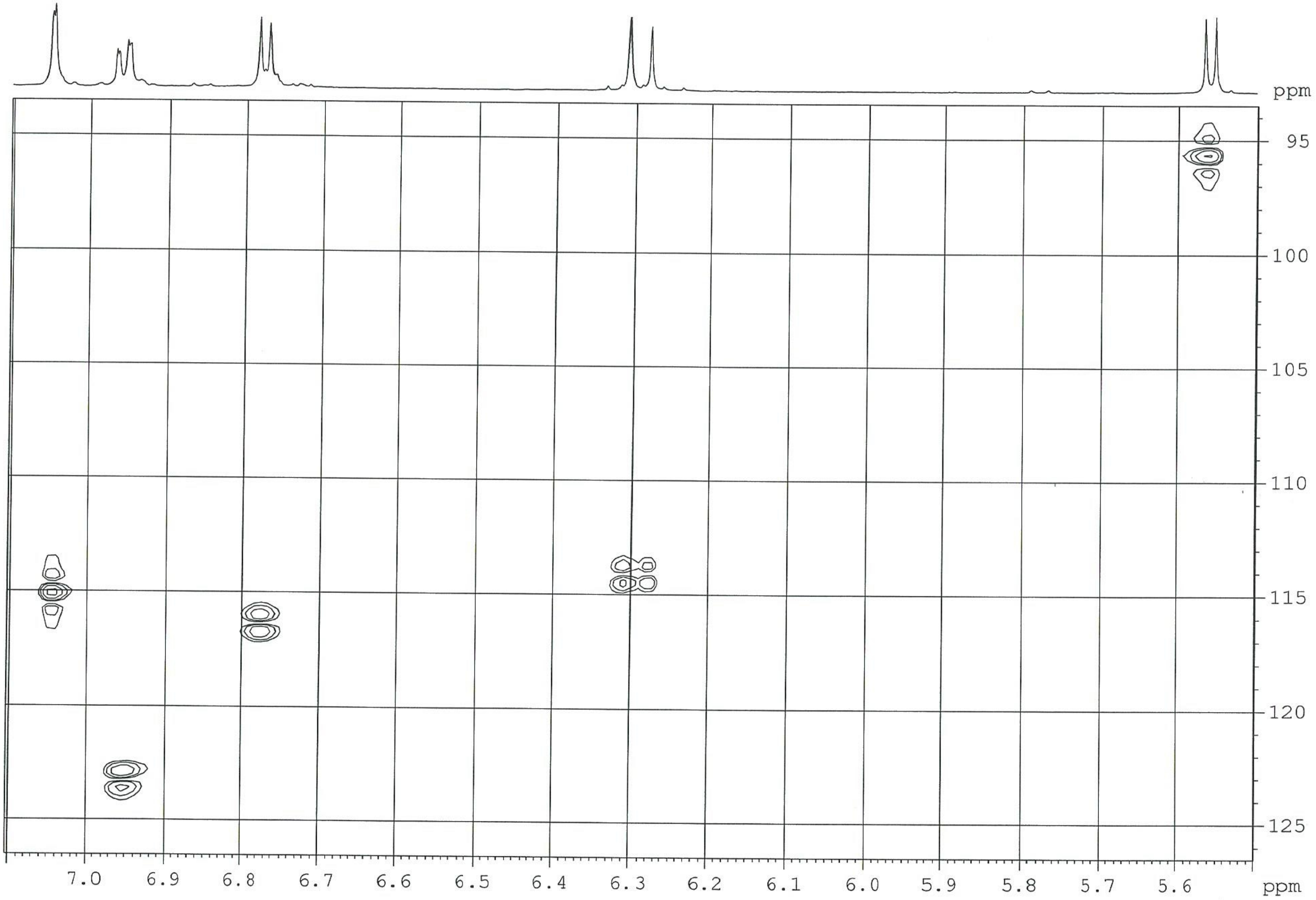

DR.SHAKIL/ RMH
ICCBS/U.O.K
HSQC

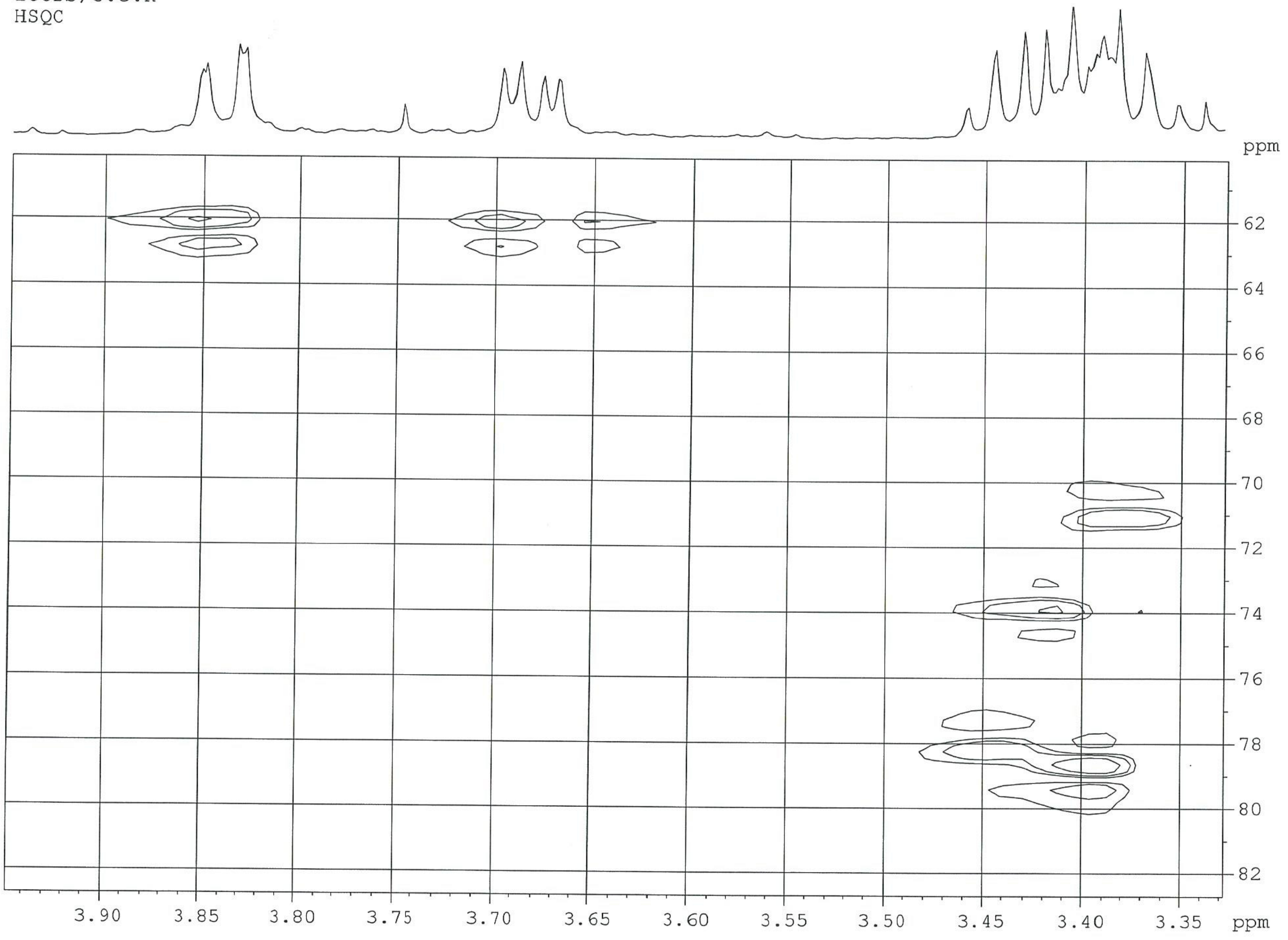

RMH-MASS

ICCBS(HEJ-LAB-101)
1/6/2015 11:22:37 AM

File: RMH
Sample: DR.SHAKIL /IAC
Instrument: JEOL JMS-600H
Inlet: Direct Probe

Date Run: 01-06-2015 (Time Run: 10:35:49)

Ionization mode: EI+

Run By: HEJ (ICCBS]

Scan: 14
Base: m/z 136; 65.2%FS TIC: 6206030

R.T.: 1.08

#Ions: 546

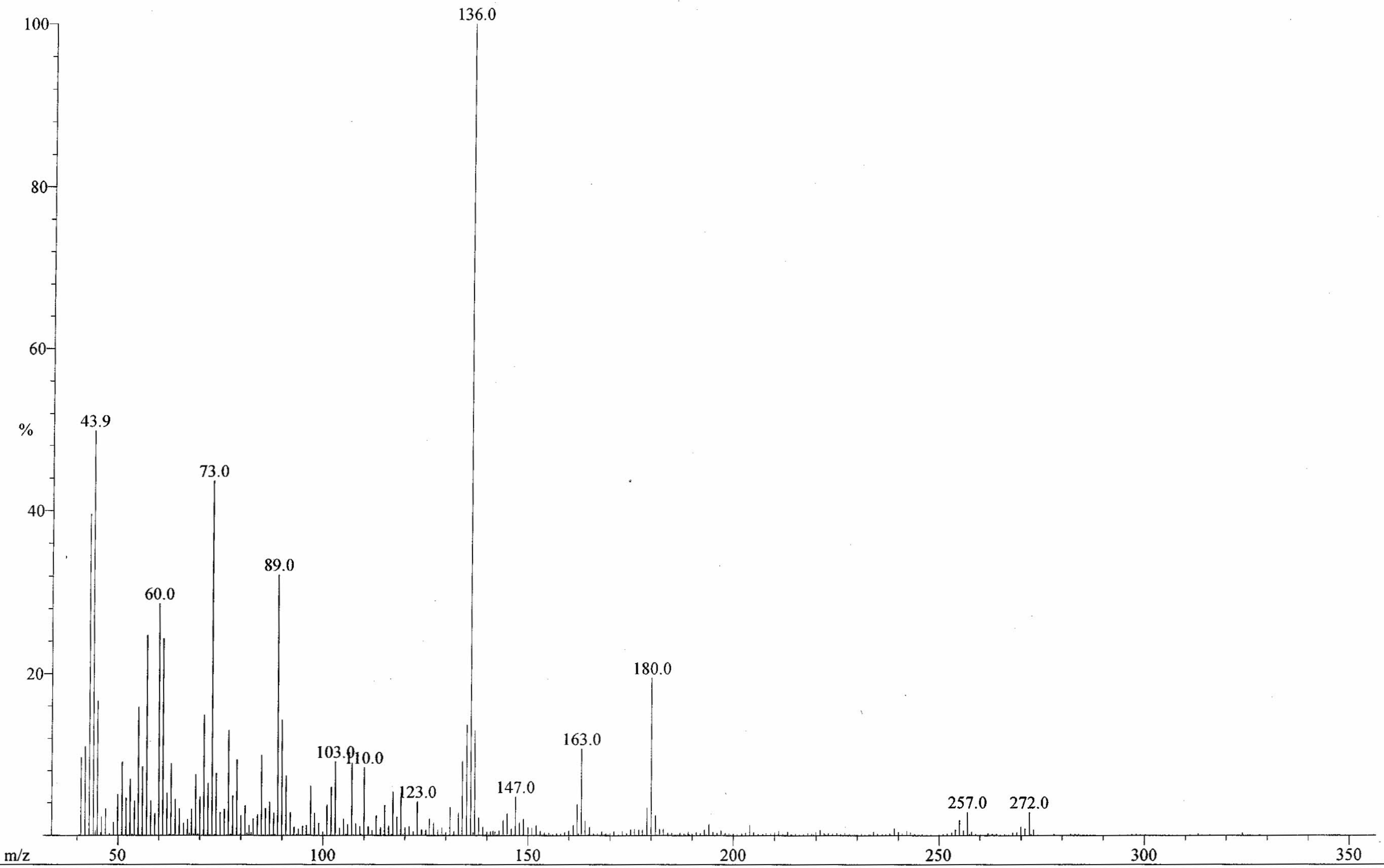

File: RMH
Date Run: 01-06-2015 (Time Run: 10:35:49)
Sample: DR.SHAKIL /IAC
Instrument: JEOL JMS-600H
Run By: HEJ (ICCBS]
Inlet: Direct Probe
Ionization mode: EI+

Scan: 14
R.T.: 1.08
Base: m/z 136; 65.2%FS TIC: 6206030
#Ions: 546

Threshold: 1.3% of Base
Displayed TIC: 6206030

Mass	%Base	Mass	%Base	Mass	%Base	Mass	%Base	Mass	%Base	Mass	%Base	Mass	%Base
41.0	9.7	56.0	8.5	70.0	4.8	85.0	9.9	103.0	9.1	131.0	3.5	164.0	1.8
41.9	11.1	57.0	24.8	71.0	14.9	86.0	3.4	105.0	2.1	133.0	2.8	179.0	3.4
43.0	39.6	58.0	4.3	72.0	6.5	87.0	4.2	106.0	1.4	134.0	9.2	180.0	19.5
43.9	49.9	59.0	2.8	73.0	43.7	88.0	2.8	107.0	9.0	135.0	13.7	181.0	2.5
45.0	16.6	60.0	28.6	74.0	7.7	89.0	32.2	108.0	1.5	136.0	100.0	194.0	1.4
45.9	2.3	61.0	24.4	75.0	2.9	90.0	14.3	110.0	8.4	137.0	13.0	255.0	1.9
46.9	3.4	62.0	5.3	76.0	3.2	91.0	7.4	112.9	2.5	138.0	2.2	257.0	2.9
49.0	1.7	63.0	8.9	77.0	13.0	92.0	2.9	115.0	3.8	144.0	1.9	272.0	2.9
49.9	5.1	64.0	4.5	78.0	4.9	95.9	1.3	117.0	5.4	145.0	2.7		
51.0	9.2	65.0	3.3	79.0	9.4	97.0	6.1	118.0	2.3	147.0	4.8		
52.0	4.7	66.0	1.6	80.0	2.5	97.9	2.8	119.0	5.2	148.0	1.6		
53.0	7.0	67.0	2.0	81.0	3.7	99.0	1.5	123.0	4.2	149.0	2.1		
54.0	4.3	68.0	3.3	83.0	2.1	100.9	3.8	126.0	2.1	161.9	3.8		
55.0	15.9	69.0	7.6	84.0	2.6	102.0	6.0	127.0	1.6	163.0	10.7		

RMH-PROTON

DR.SHAKIL/RMH PURE/CD3OD
ICCBS/U.O.K

AVANCE AV-500
LAB NO: 109B

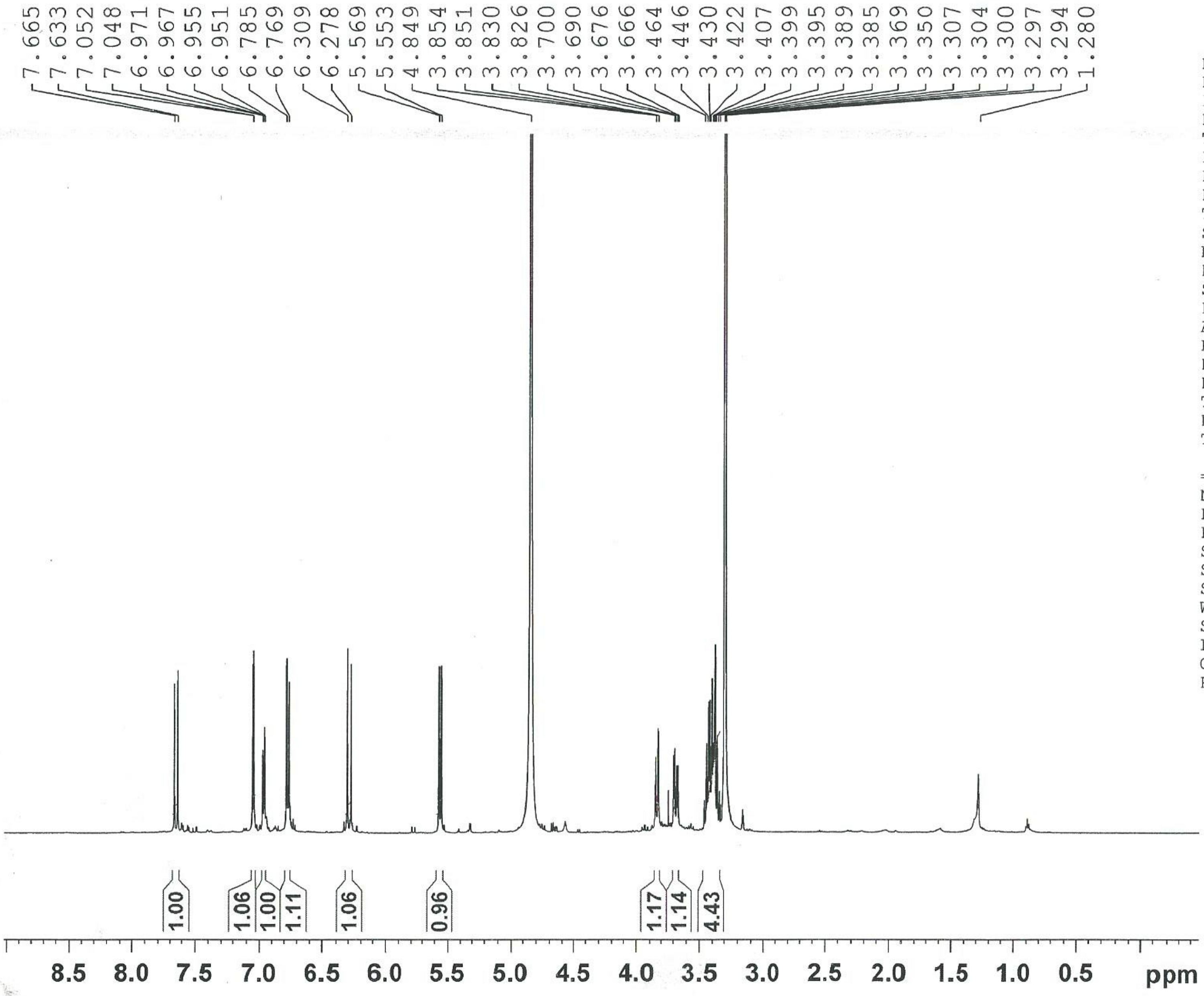

NAME	dec01-14	
EXPNO	6	
PROCNO	1	
Date_	20141201	
Time	15.12	
INSTRUM	spect	
PROBHD	5 mm BBI 1H/D-	
PULPROG	zg30	
TD	65536	
SOLVENT	MeOD	
NS	128	
DS	0	
SWH	10330.578	Hz
FIDRES	0.157632	Hz
AQ	3.1720407	sec
RG	456.1	
DW	48.400	usec
DE	6.50	usec
TE	297.9	K
D1	2.00000000	sec
TD0	1	

======== CHANNEL f1 ========

NUC1	1H	
P1	8.00	usec
PL1	-1.00	dB
SFO1	500.1335009	MHz
SI	32768	
SF	500.1300158	MHz
WDW	EM	
SSB	0	
LB	0.30	Hz
GB	0	
PC	1.00	

DR.SHAKIL/RMH PURE/CD3OD
ICCBS/U.O.K

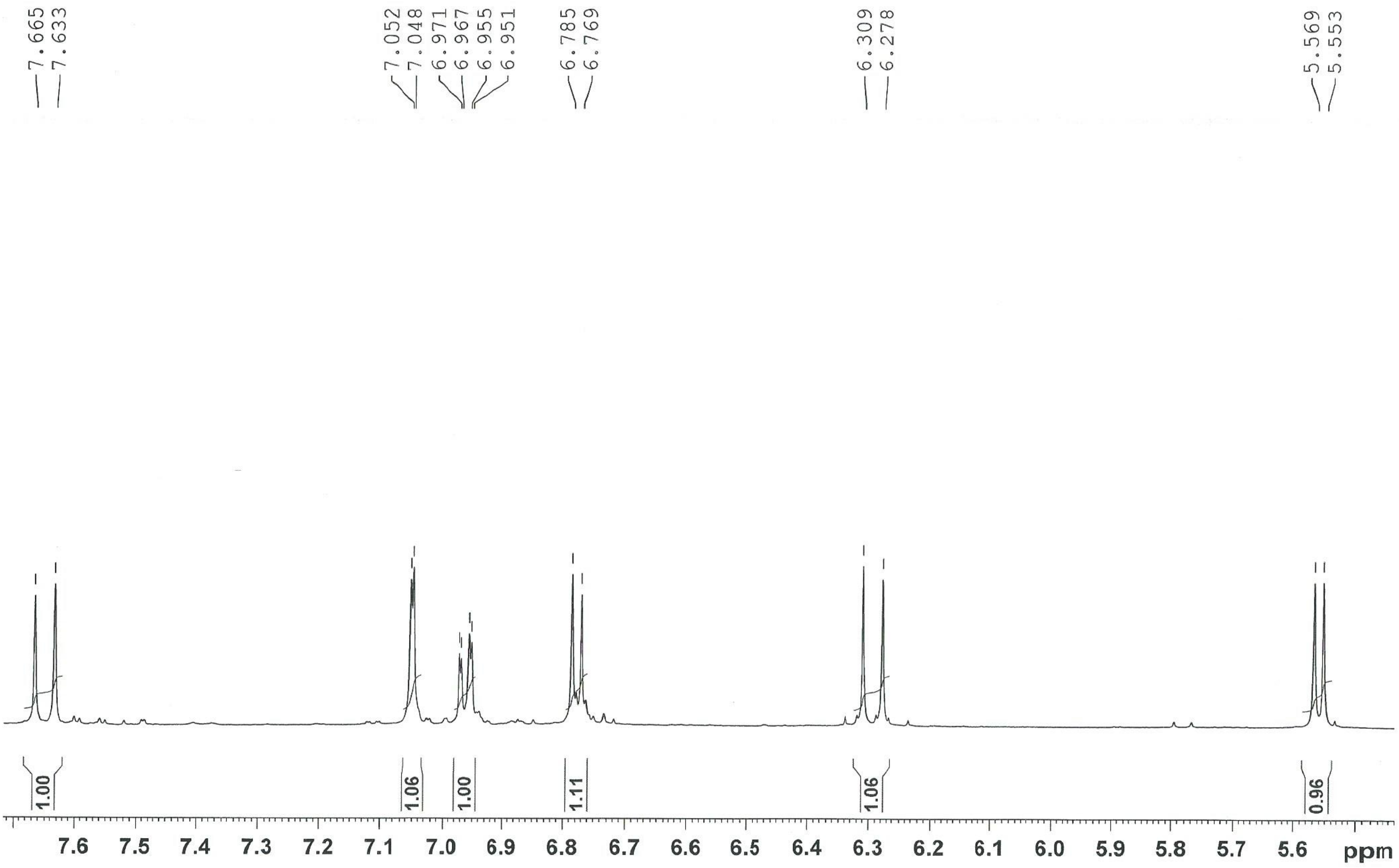

DR.SHAKIL/RMH PURE/CD3OD
ICCBS/U.O.K

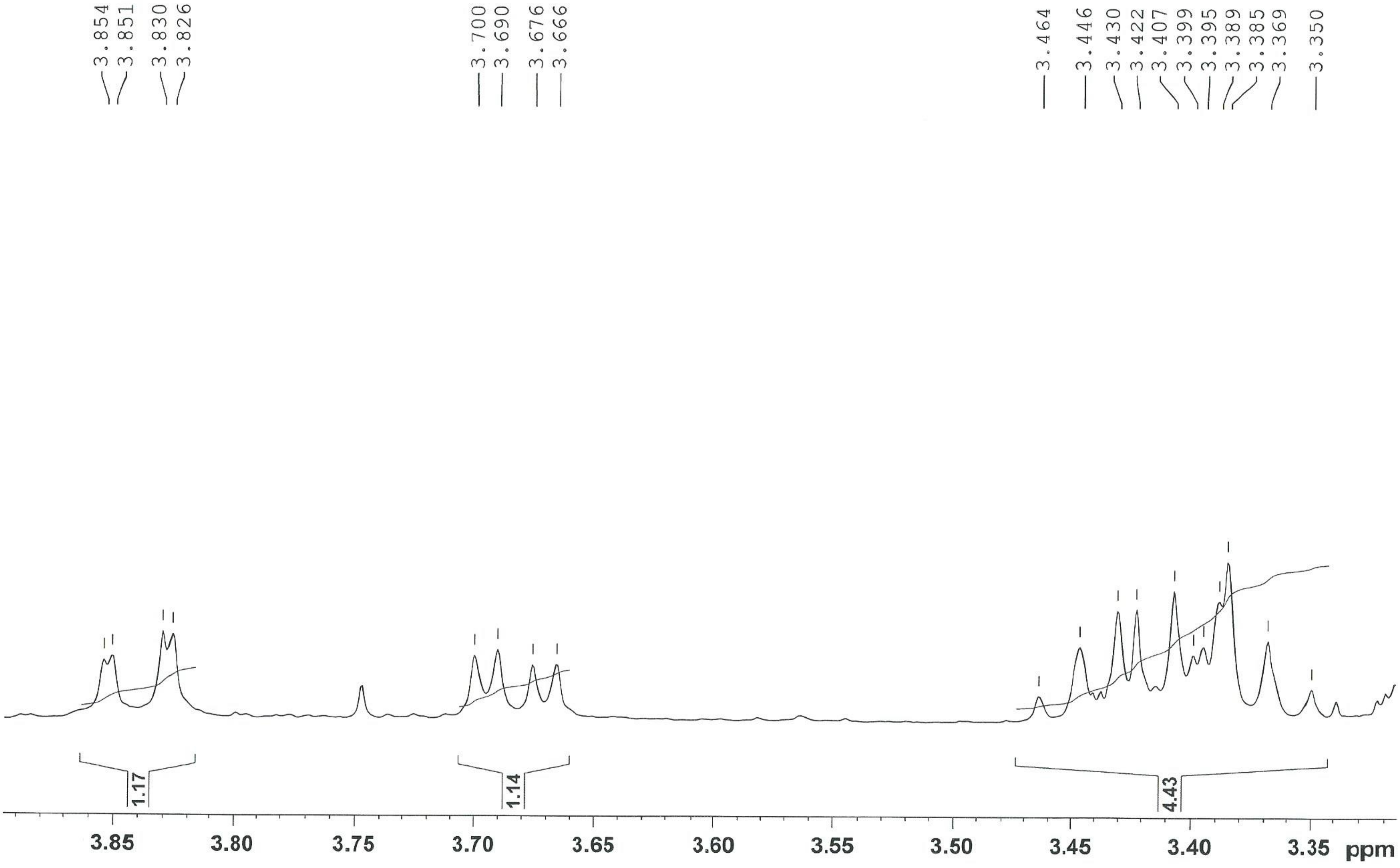

RMI-BB

DR.SHAKIL / Rmi

BB

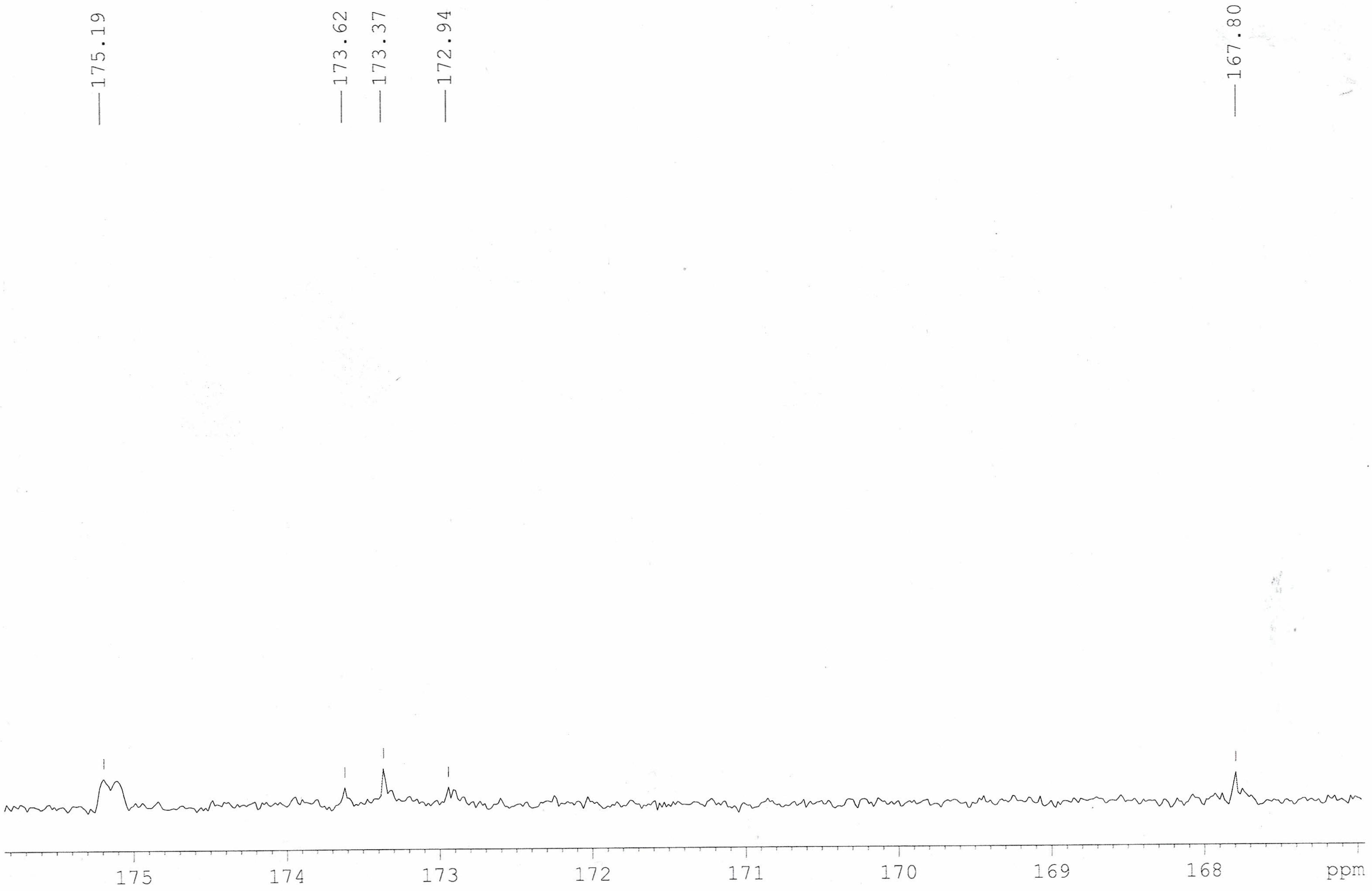

DR.SHAKIL / Rmi
BB

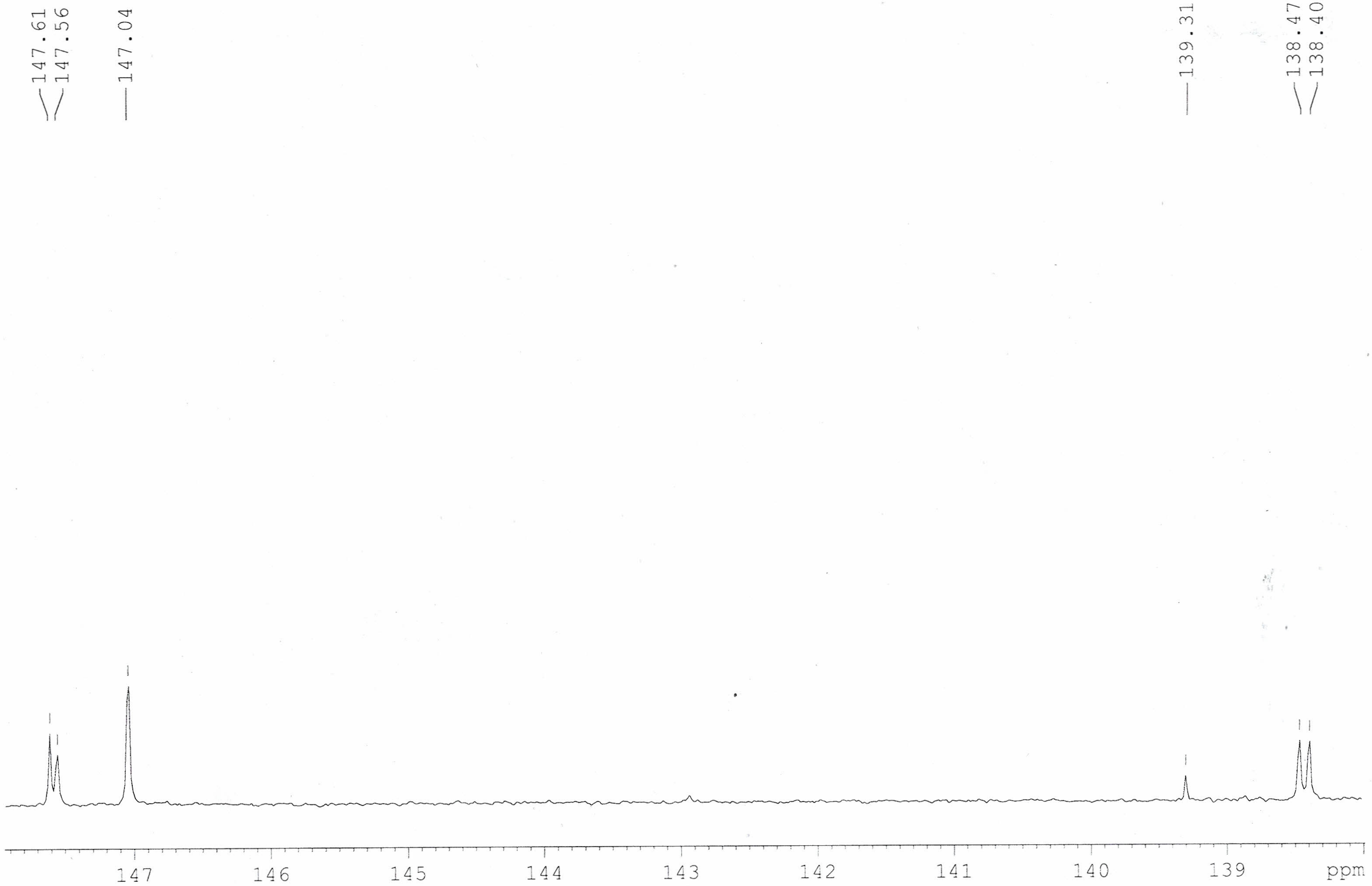

DR.SHAKIL / Rmi
BB

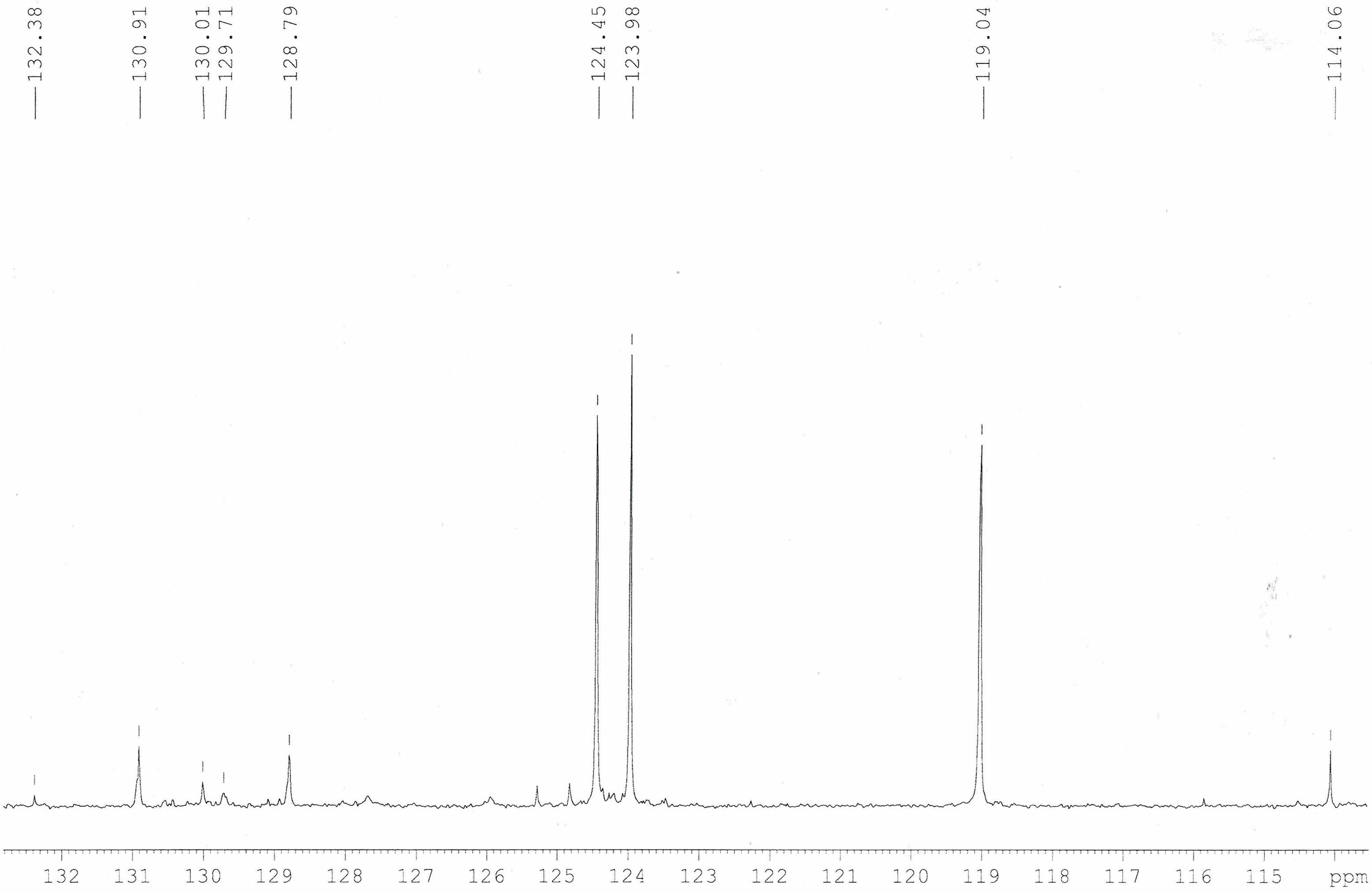

DR.SHAKIL / Rmi
BB

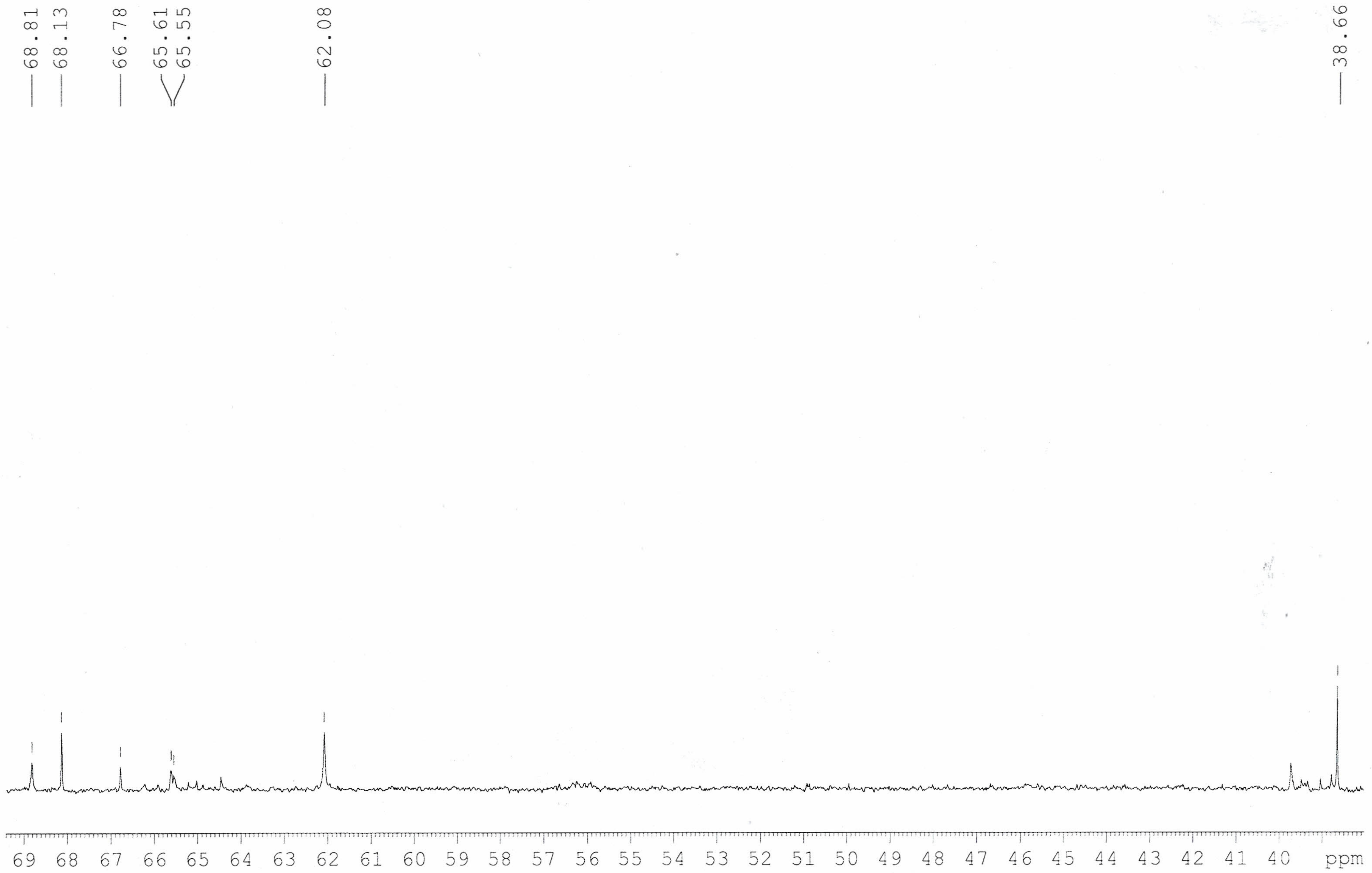

DR.SHAKIL / Rmi
BB

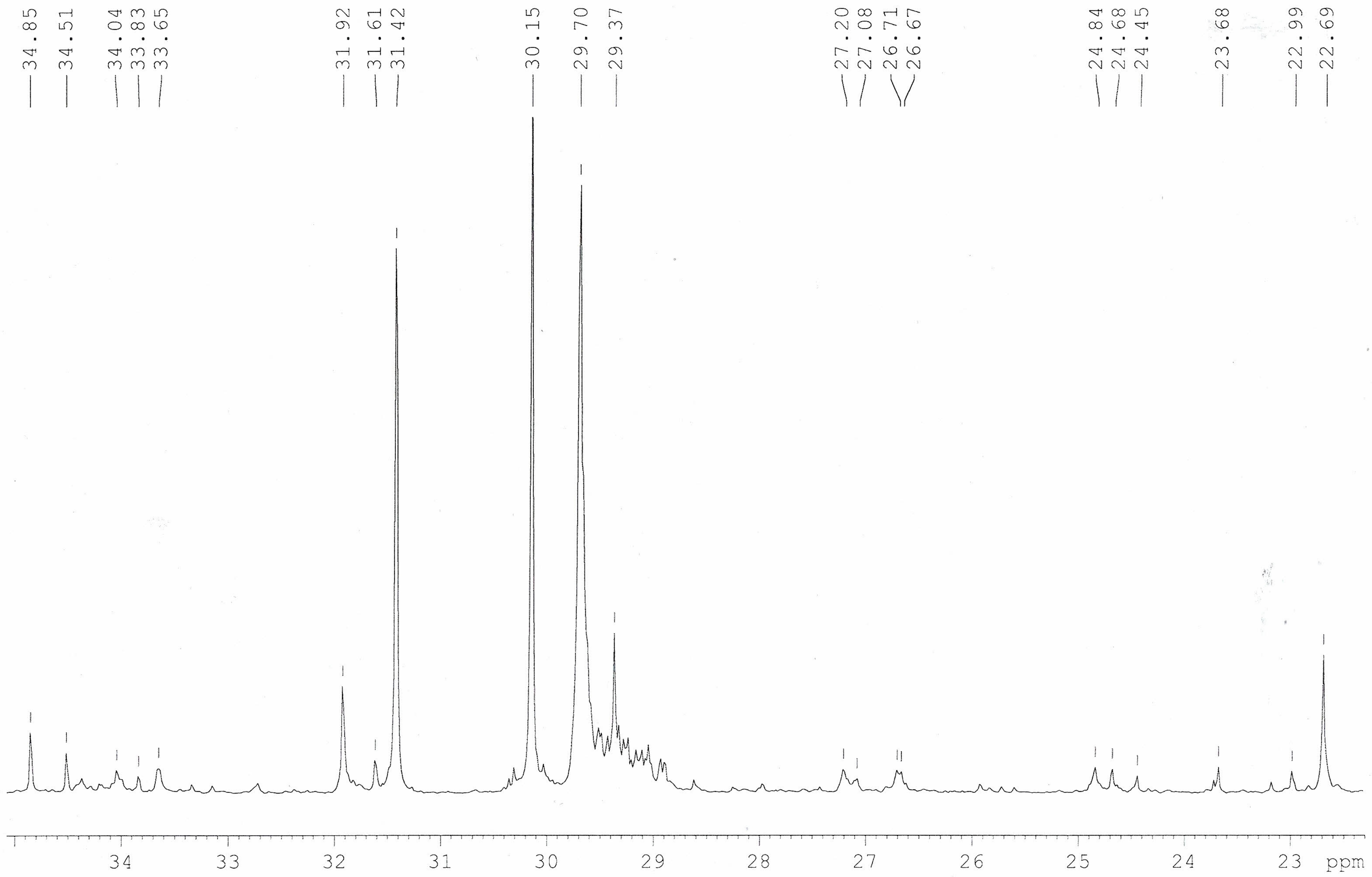

DR.SHAKIL / Rmi
BB

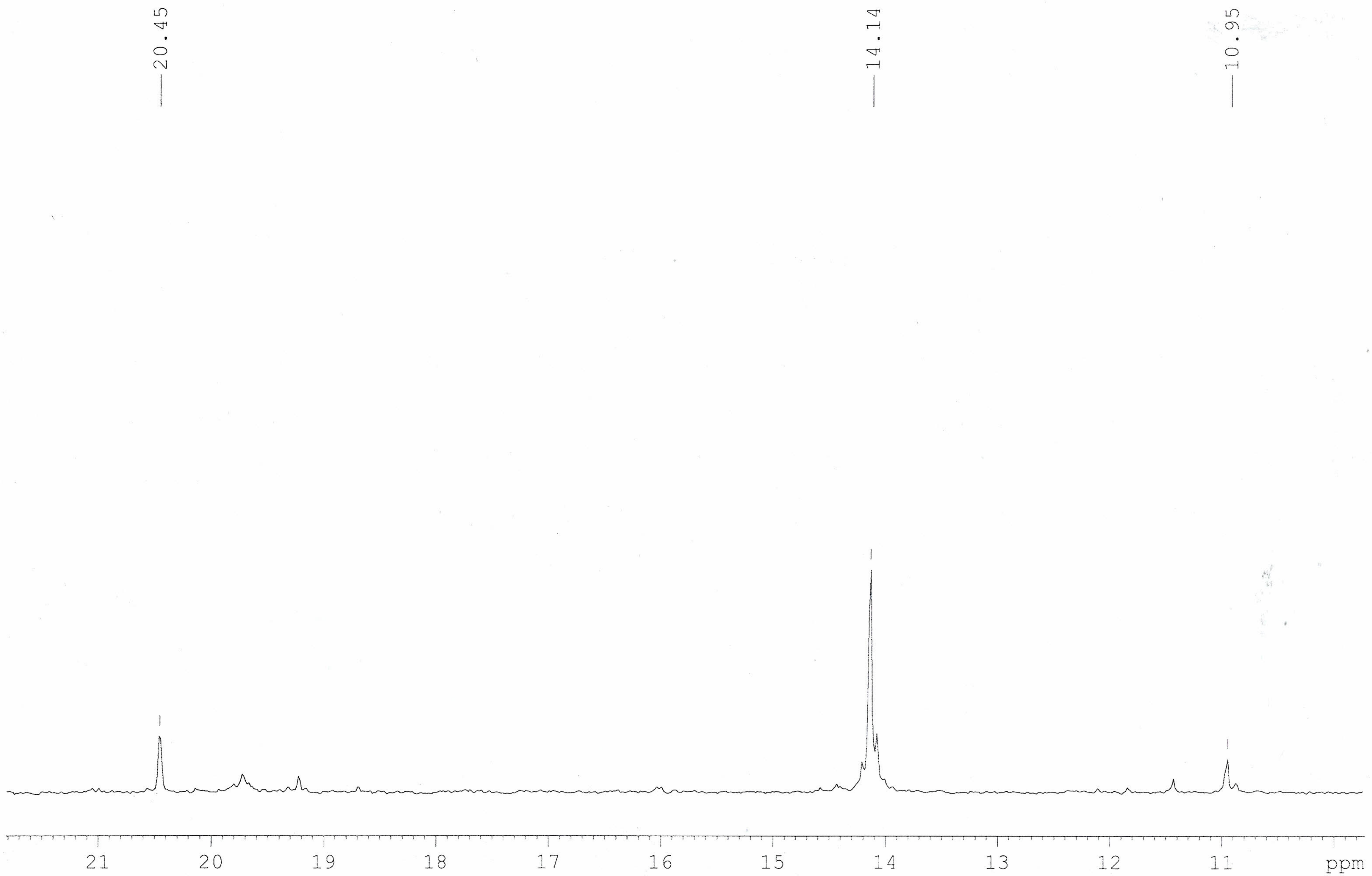

RMI-HMBC

DR.SHAKIL / Rmi
HMBC

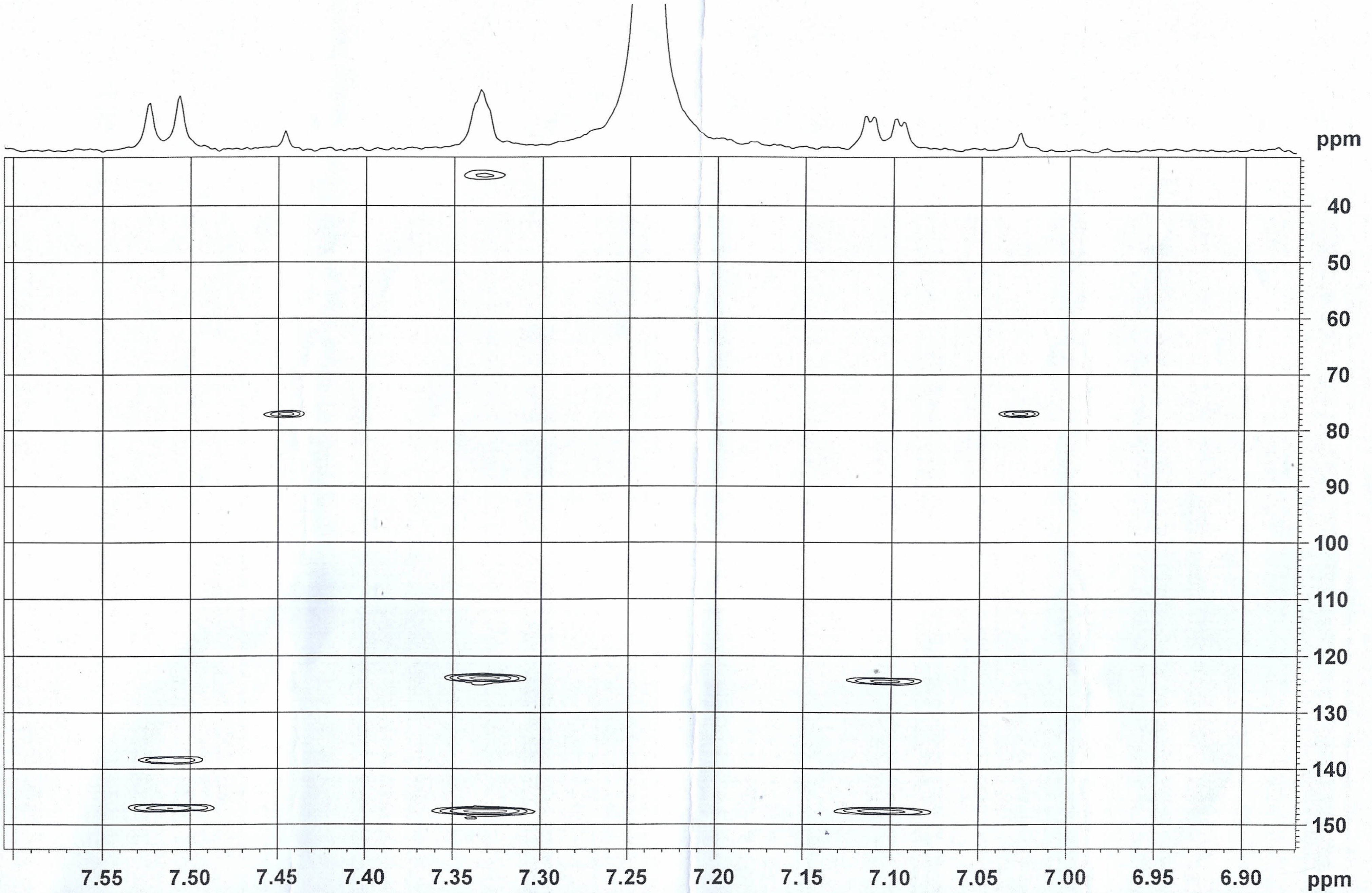

DR.SHAKIL / Rmi
HMBC

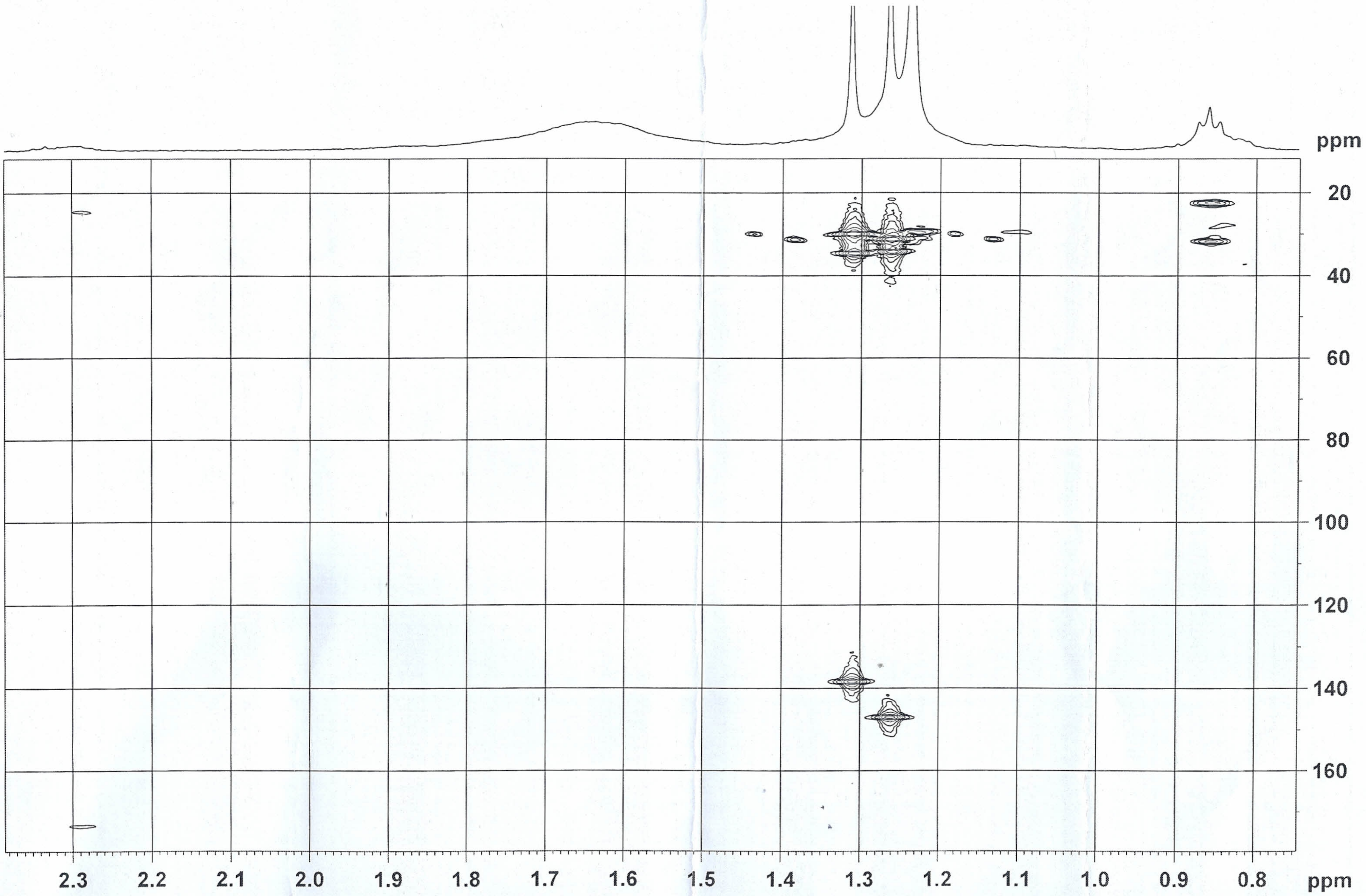

DR.SHAKIL / Rmi
HMBC

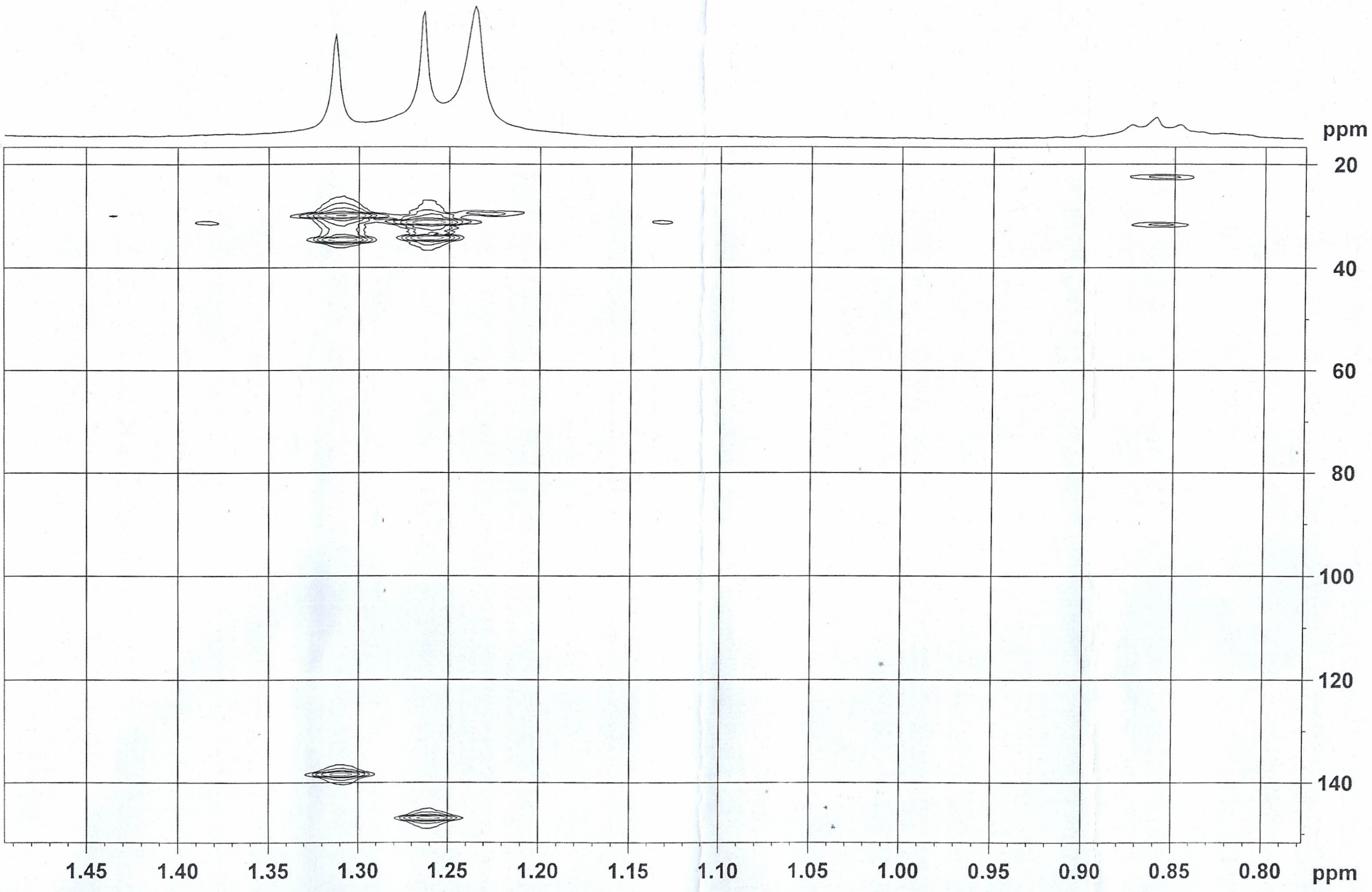

RMI-HSQC

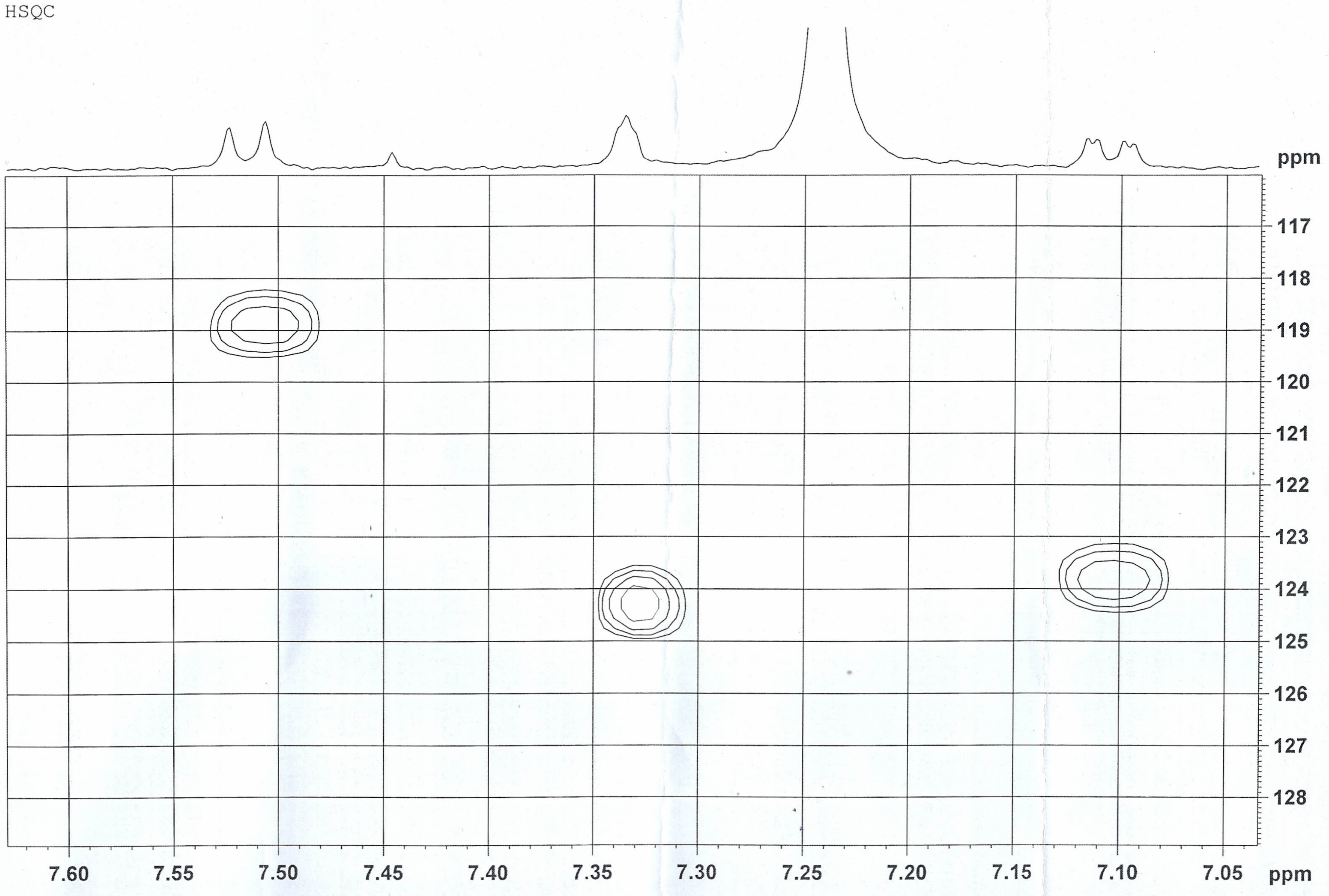

HSQC
ppm
117
118
119
120
121
122
123
124
125
126
127
128
7.60
7.55
7.50
7.45
7.40
7.35
7.30
7.25
7.20
7.15
7.10
7.05
ppm

DR.SHAKIL / Rmi
HSQC

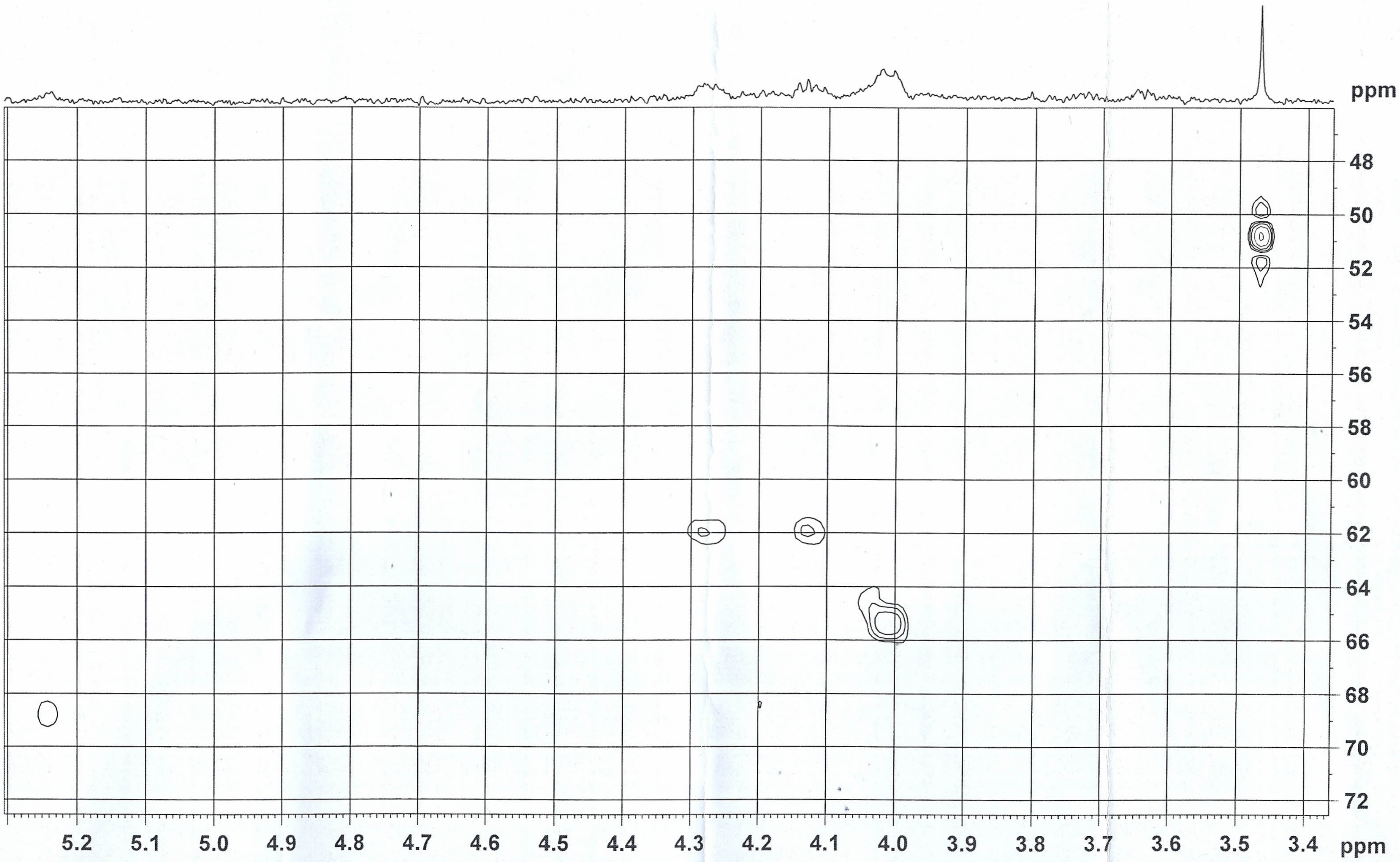

DR.SHAKIL / Rmi
HSQC

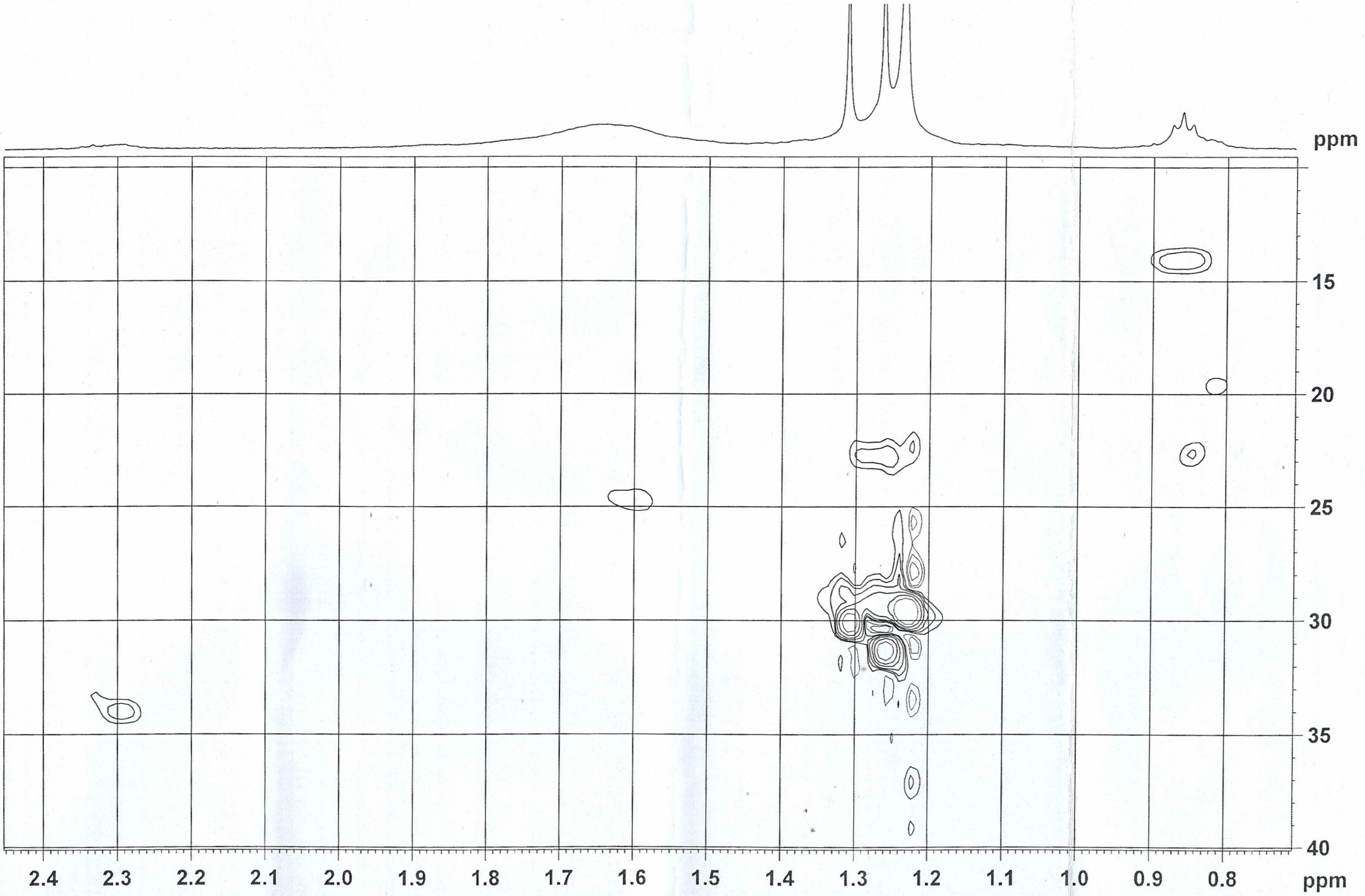

DR.SHAKIL / Rmi
HSQC

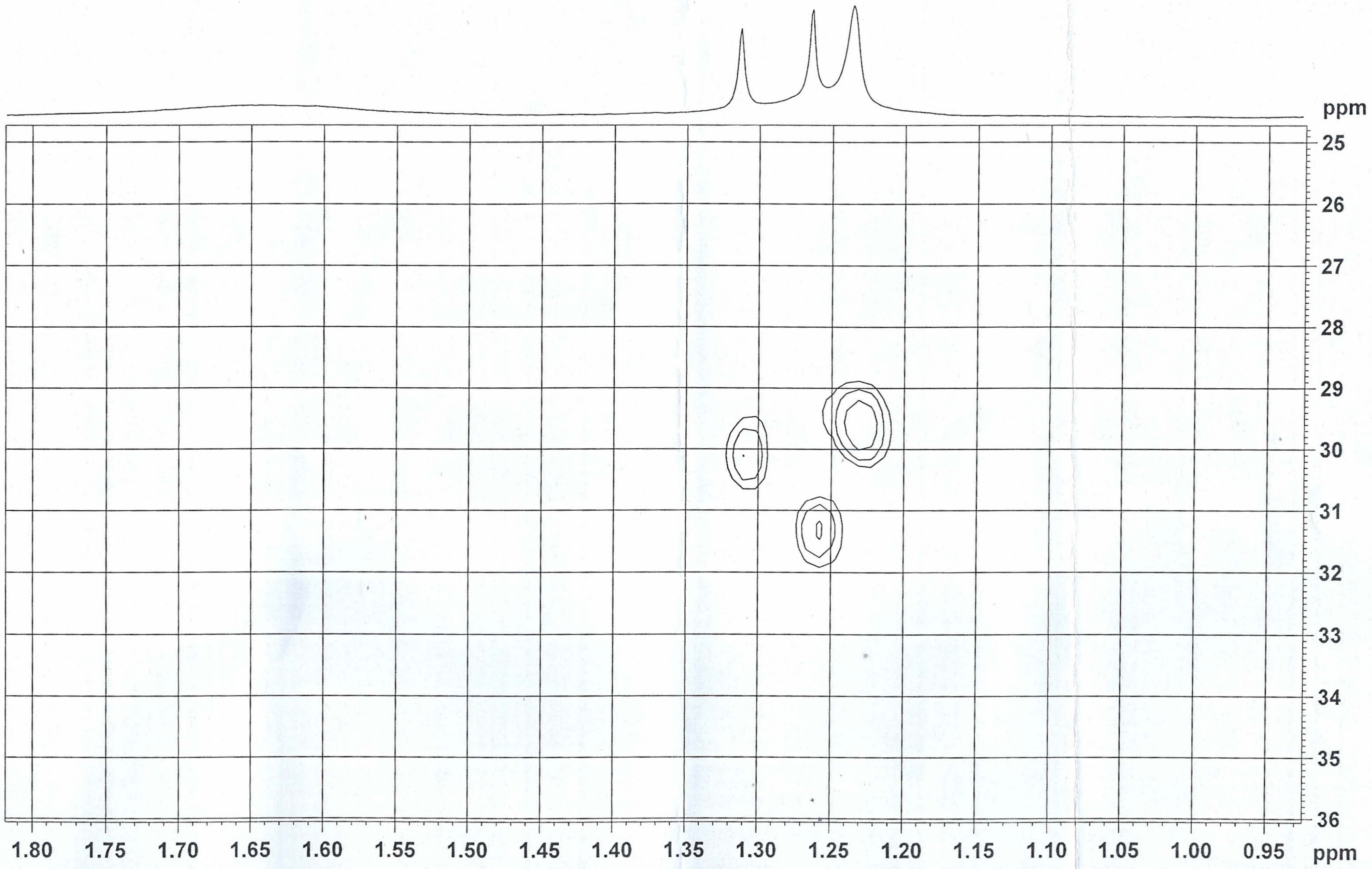

RMI-MASS

File Name	:	d:\mswin\data\rmi.mss
Creation Date/Time	:	21.05.15 at 20:32:08
File Type	:	Lo-Res Mass Data (Centroid)
File Source	:	Acquired on MASPEC system [msw/A091]
File Title	:	DR. SHAKIL
Operator	:	Barkat Ali
Instrument	:	MAT312

SCAN TEXT.Filter=[Int:0.5%.].
Scan 3-2:03. Sorted on M/z (ascending). Entries=66.

---M/z--	---Int.---		---%age---
51.0	1516		1.97
53.1	2147		2.79
54.0	417		0.54
55.1	55749		72.39
56.1	16684		21.66
57.0	77016		100.00
58.1	8267		10.73
59.0	679		0.88
60.0	19624		25.48
61.0	449		0.58
65.0	619		0.80
67.0	12587		16.34
68.0	2478		3.22
68.9	31957	m	41.49
69.0	34398	m	44.66
70.0	10688		13.88
71.1	38827		50.41
73.0	11806		15.33
74.0	1009		1.31
77.0	2648		3.44
79.0	3195		4.15
81.0	13571		17.62
82.0	5638		7.32
83.0	37937		49.26
84.0	3120		4.05
85.0	30155		39.15
87.0	1407		1.83
91.0	13082		16.99
93.0	780		1.01
95.1	9095		11.81
96.1	2578		3.35
97.1	14507		18.84
98.1	1536		1.99
99.1	6381		8.29
105.1	2957		3.84
107.1	2487		3.23
109.1	1368		1.78
111.1	2098		2.72
113.1	1706		2.22
113.1	1086		1.41
113.1	856		1.11
115.1	1447		1.88
116.1	610		0.79

---M/z--	---Int.---		---%age---
118.9	2201		2.86
129.0	2745		3.56
129.1	11873		15.42
130.8	411		0.53
131.0	4058		5.27
146.9	2922		3.79
149.0	875		1.14
175.0	1163		1.51
191.0	21242		27.58
290.9	2750		3.57
315.9	26282	m	34.13
316.5	5433		7.05
366.8	3748		4.87
423.0	2177		2.83
479.0	2562		3.33
534.9	3271		4.25
591.2	6987		9.07
647.0	62597	m	81.28
648.0	43147	m	56.02
649.0	9641		12.52
662.1	39583	m	51.40
663.1	36209	m	47.01
664.3	4453		5.78

---M/z--	---Int.---	---%age---

File Name	:	d:\mswin\data\rmi.mss
Creation Date/Time	:	21.05.15 at 20:32:08
File Type	:	Lo-Res Mass Data (Centroid)
File Source	:	Acquired on MASPEC system [msw/A091]
File Title	:	DR. SHAKIL
Operator	:	Barkat Ali
Instrument	:	MAT312

SCAN GRAPH. Flagging=M/z.

Scan 3-2:03. Entries=75. 100% Int.=77016.

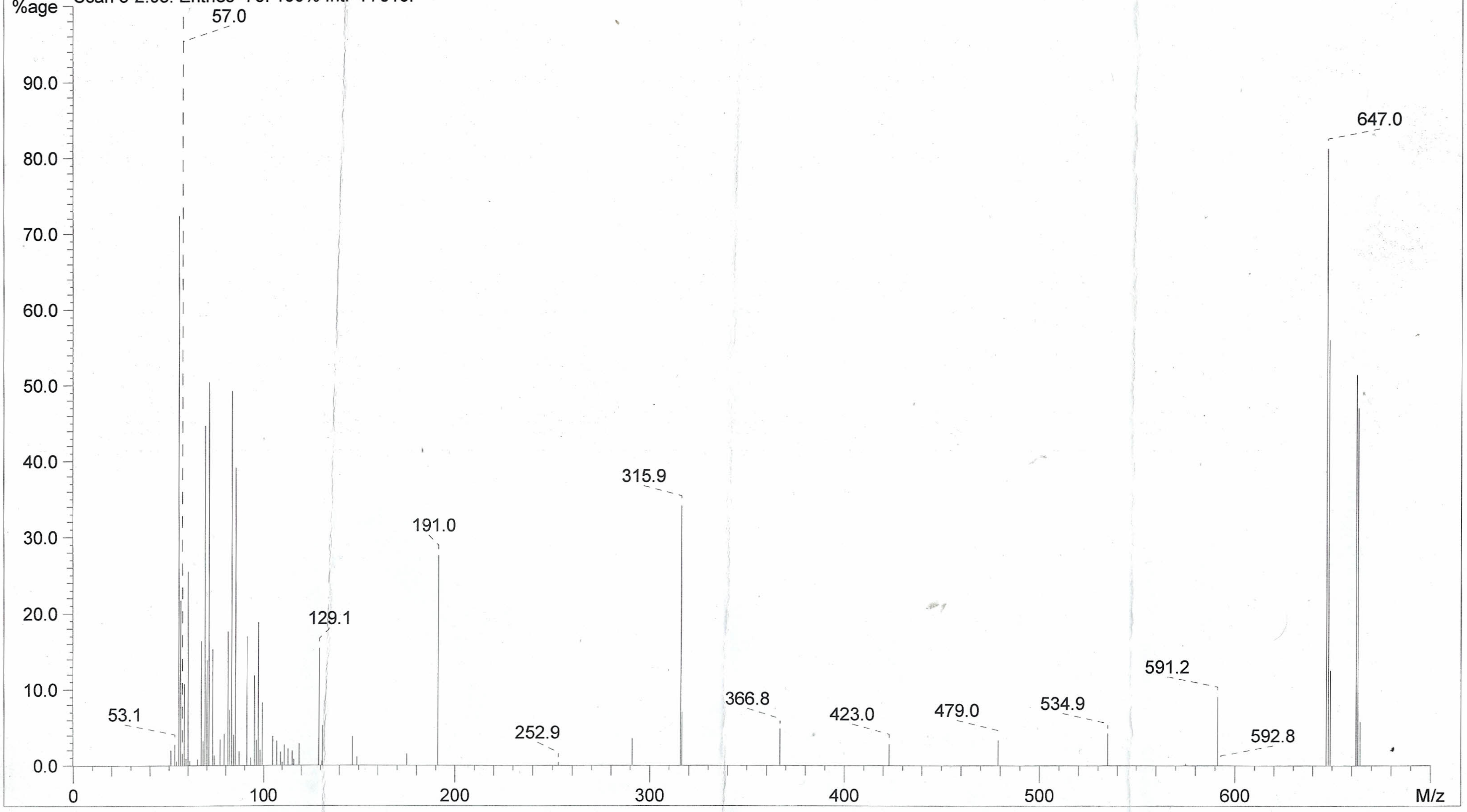

RMI-PROTON

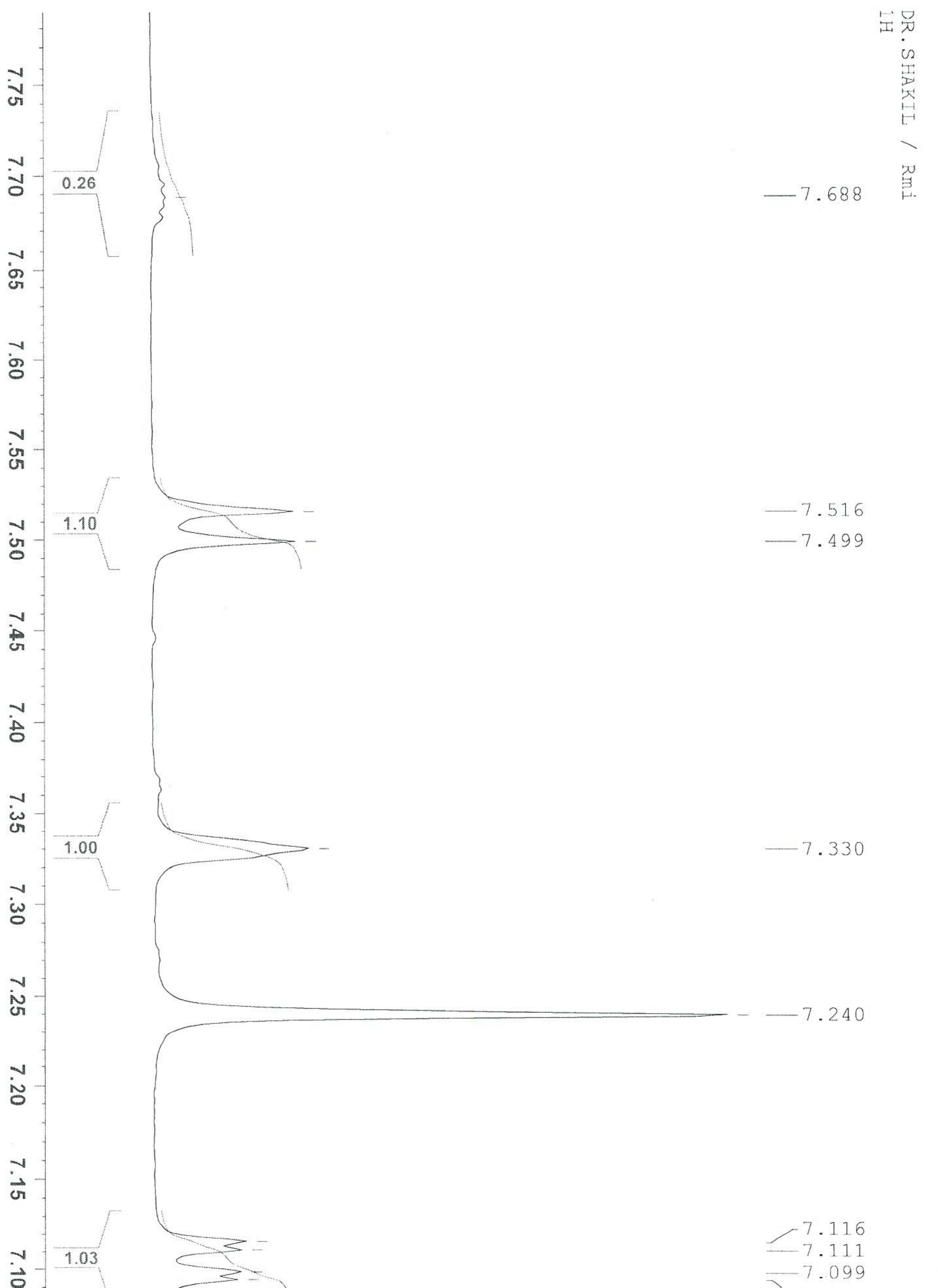
DR.SHAKIL / Rmi
1H
7.688
7.516
7.499
7.330
7.240
7.116
7.111
7.099
0.26
1.10
1.00
1.03
7.75
7.70
7.65
7.60
7.55
7.50
7.45
7.40
7.35
7.30
7.25
7.20
7.15
7.10

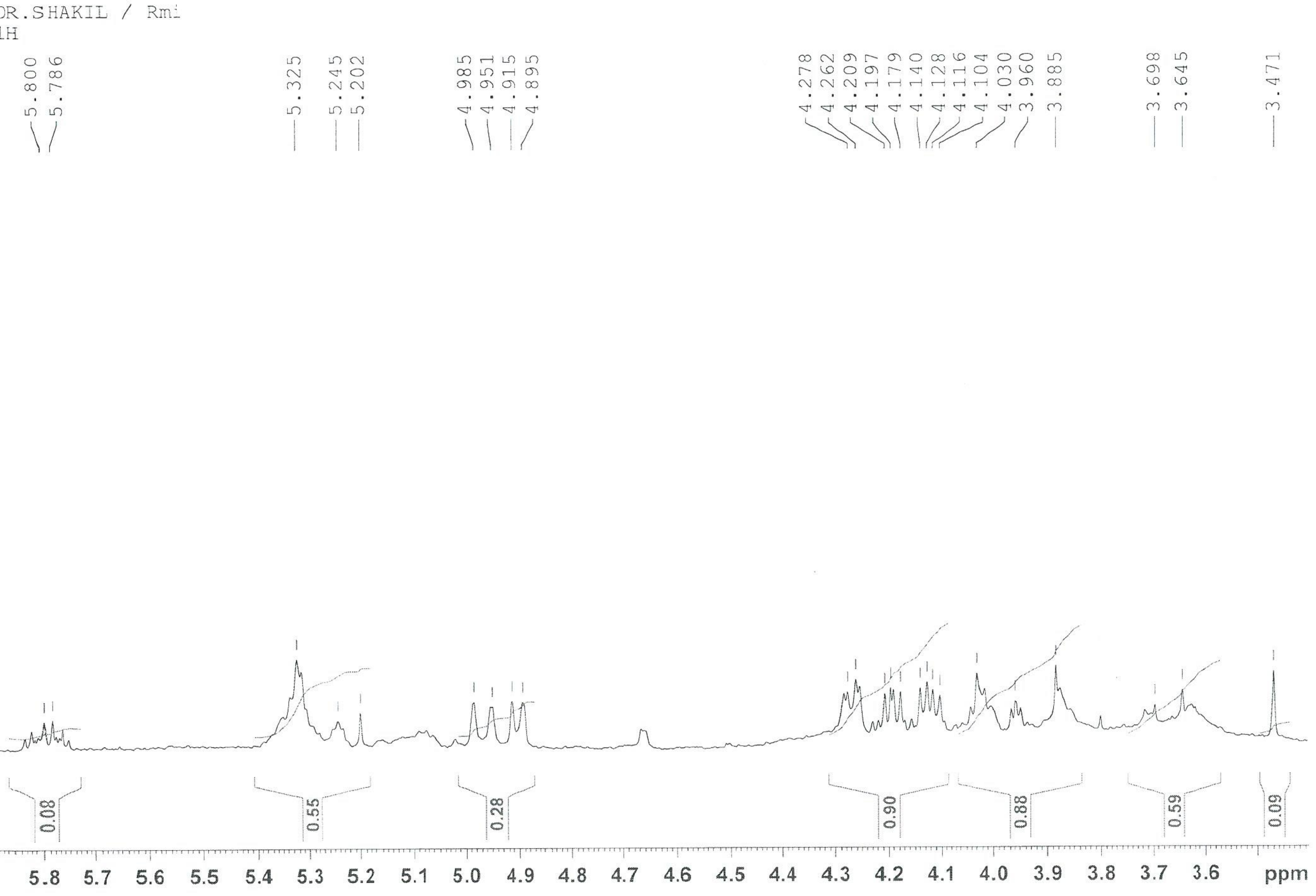

DR.SHAKIL / Rmi
1H
5.800
5.786
5.325
5.245
5.202
4.985
4.951
4.915
4.895
4.278
4.262
4.209
4.197
4.179
4.140
4.128
4.116
4.104
4.030
3.960
3.885
3.698
3.645
3.471
0.08
0.55
0.28
0.90
0.88
0.59
0.09
5.8 5.7 5.6 5.5 5.4 5.3 5.2 5.1 5.0 4.9 4.8 4.7 4.6 4.5 4.4 4.3 4.2 4.1 4.0 3.9 3.8 3.7 3.6 ppm

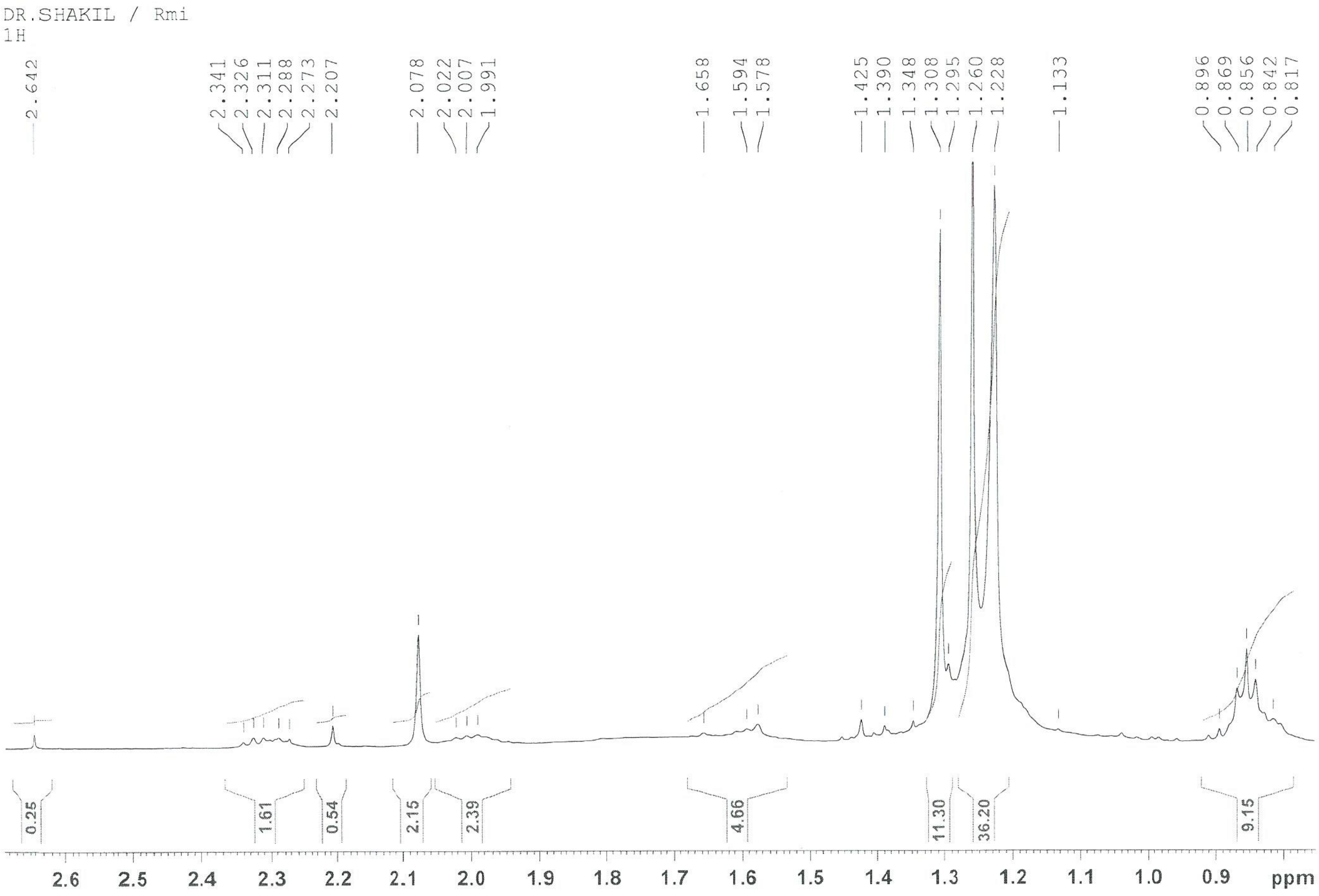
DR.SHAKIL / Rmi
1H
2.642
2.341
2.326
2.311
2.288
2.273
2.207
2.078
2.022
2.007
1.991
1.658
1.594
1.578
1.425
1.390
1.348
1.308
1.295
1.260
1.228
1.133
0.896
0.869
0.856
0.842
0.817
0.25
1.61
0.54
2.15
2.39
4.66
11.30
36.20
9.15
2.6
2.5
2.4
2.3
2.2
2.1
2.0
1.9
1.8
1.7
1.6
1.5
1.4
1.3
1.2
1.1
1.0
0.9
ppm

Wed Jan 07 15:25:13 CET 2015
